# Specification and spatial arrangement of cells in the germline stem cell niche of the *Drosophila* ovary depend on the Maf transcription factor Traffic jam

**DOI:** 10.1371/journal.pgen.1006790

**Published:** 2017-05-19

**Authors:** Trupti Panchal, Xi Chen, Ekaterina Alchits, Youjin Oh, James Poon, Jane Kouptsova, Frank A. Laski, Dorothea Godt

**Affiliations:** 1 Department of Cell and Systems Biology, University of Toronto, Toronto, Ontario, Canada; 2 Department of Molecular, Cell, and Developmental Biology, University of California Los Angeles, Los Angeles, California, United States of America; Stowers Institute for Medical Research, UNITED STATES

## Abstract

Germline stem cells in the *Drosophila* ovary are maintained by a somatic niche. The niche is structurally and functionally complex and contains four cell types, the escort, cap, and terminal filament cells and the newly identified transition cell. We find that the large Maf transcription factor Traffic jam (Tj) is essential for determining niche cell fates and architecture, enabling each niche in the ovary to support a normal complement of 2–3 germline stem cells. In particular, we focused on the question of how cap cells form. Cap cells express Tj and are considered the key component of a mature germline stem cell niche. We conclude that Tj controls the specification of cap cells, as the complete loss of Tj function caused the development of additional terminal filament cells at the expense of cap cells, and terminal filament cells developed cap cell characteristics when induced to express Tj. Further, we propose that Tj controls the morphogenetic behavior of cap cells as they adopted the shape and spatial organization of terminal filament cells but otherwise appeared to retain their fate when Tj expression was only partially reduced. Our data indicate that Tj contributes to the establishment of germline stem cells by promoting the cap cell fate, and controls the stem cell-carrying capacity of the niche by regulating niche architecture. Analysis of the interactions between Tj and the Notch (N) pathway indicates that Tj and N have distinct functions in the cap cell specification program. We propose that formation of cap cells depends on the combined activities of Tj and the N pathway, with Tj promoting the cap cell fate by blocking the terminal filament cell fate, and N supporting cap cells by preventing the escort cell fate and/or controlling the number of cap cell precursors.

## Author summary

Establishment and maintenance of stem cells often depends on associated niche cells. The germline stem cell niche of the *Drosophila* ovary has been a long-standing model for the analysis of the interactions between stem cells and niche cells. Surprisingly little is known, however, about the mechanisms that pattern this niche, leading to the specification of different niche cell types and to their distinct arrangement around the stem cells. The observation that Tj is expressed at different levels in the different cell types of the niche motivated us to ask what contribution this transcription factor makes to the formation of the niche. Our data suggest that Tj activity is needed for the presence of escort cells and for the correct specification of cap cells but appears to be dispensable for the formation of terminal filament cells in the germline stem cell niche. Moreover, our analysis indicates that the establishment of the cap cell fate depends on the cooperation between Tj and the N signaling pathway. We conclude that Tj regulates the germline stem cell carrying capacity of the niche by controlling the fate and the spatial arrangement of niche cells.

## Introduction

Stem cells retain the capacity for development in differentiated organisms, which is important for tissue growth, homeostasis and regeneration, and for long-term reproductive capability. Stem cells are often associated with a specialized microenvironment, a niche that is essential for the formation, maintenance, and self-renewal of stem cells by preventing cell differentiation and controlling rate and mode of cell division [1,2]. The niche for the germline stem cells (GSCs) in *Drosophila* serves as an important model for the analysis of interactions between niche and stem cells [1,3–5]. The astounding fecundity of *Drosophila* females that can lay dozens of eggs per day over several weeks depends on approximately 100 GSCs that are sustained by 40 stem cell niches. To understand the formation and maintenance of these GSCs, it is important to understand how stem cell niches form and how they function.

**Fig 1:**
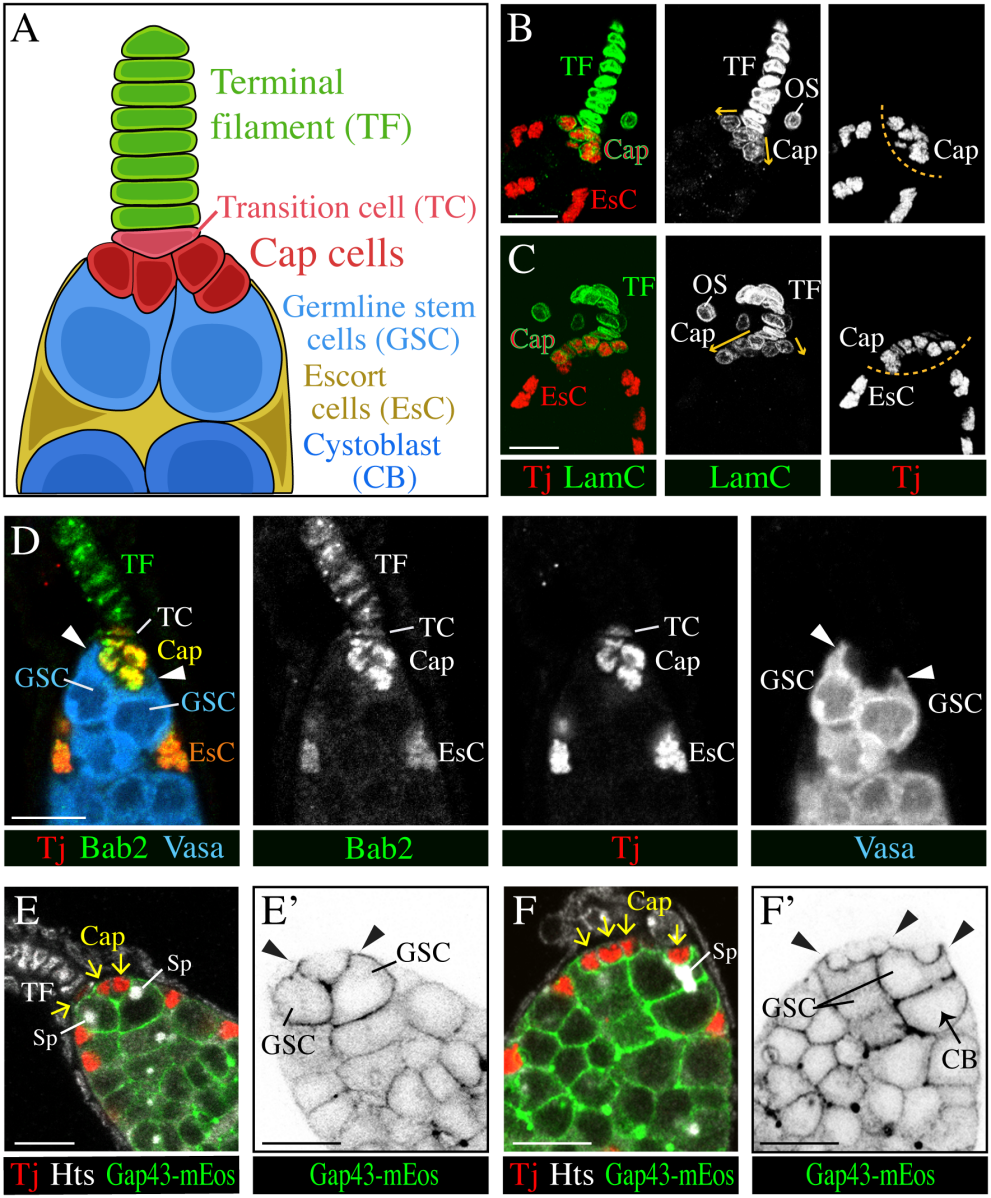
Tj expression and tissue organization in the wild-type GSC niche. (**A**) Schematic drawing of the GSC niche. (**B-C**) Cap cells can form a symmetric (B) or asymmetric cluster (C) as indicated by arrows. A broad gap is visible between the cluster of tightly packed cap cell nuclei and the nuclei of escort cells (EsC), as indicated by a dashed line. Lamin C (LamC) staining of the nuclear envelope (green) is strong in TF cells, weak in cap cells (and surrounding ovariole sheath cells (OS)), and not detectable in escort cells. (**D**) GSCs, marked by Vasa (blue) form protrusions (arrowheads) around the cap cell cluster (yellow). A transition cell (TC) connects the cap cell cluster to the TF (green). Nuclei of escort cells (EsC, orange) sit posterior to the GSCs. Tj staining (red) is very strong in escort cells, strong in cap cells, weak in the transition cell, and not detectable in the TF. Bab2 (green) is stronger expressed in cap cells than in other somatic cells. (**E-F'**) GSCs have plasma membrane protrusions, marked by GAP43-mEos (arrowheads in E',F') that partially envelop the Tj-positive (red) cap cells (arrows in E,F). Spectrosomes (Sp) of GSCs are highlighted by Hu li tai shao (Hts) (white). (F,F') A GSC is connected to its daughter cystoblast via an open ring canal. CB, pre-cystoblast/cystoblast. Anterior is up. Scale bars: 10 μm.

The GSC niche of the *Drosophila* ovary consists of three somatic cell types: cap cells, escort cells, and terminal filament (TF) cells (Fig 1A). GSCs are anchored to cap cells by DE-cadherin-mediated adhesion and require close proximity to cap cells to retain stem cell character [6–8]. Cap cells secrete the BMP homolog Decapentaplegic (Dpp), activating the TGFß signaling pathway in adjacent GSCs [9], which leads to the repression of the germline differentiation factor Bag-of-Marbles (Bam) [10,11]. Through Hedgehog (Hh) signaling, cap cells also appear to stimulate escort cells to secrete Dpp [12]. The combined pool of Dpp from cap and escort cells, together with mechanisms that concentrate Dpp in the extracellular space around GSCs [13], promotes the maintenance of 2–3 GSCs, whereas the adjacent GSC daughter cells that have lost the contact to cap cells will enter differentiation as cystoblasts [3,4]. In contrast, TFs are not in direct contact with GSCs but serve important functions in the development and probably also in the maintenance and function of GSC niches [14].

Formation of GSC niches begins with the progressive assembly of TFs by cell intercalation during the 3rd larval instar [15–17]. The process of TF cell specification is not understood but might start in 2nd instar when the first TF precursor cells appear to leave the cell cycle [18,19]. TF morphogenesis depends on the Bric à brac transcriptional regulators that control the differentiation of TF cells and their ability to form cell stacks [15,16,20], and involves the Ecdysone Receptor (EcR) [21,22], Engrailed [23], Cofilin [24], and Ran-binding protein M (RanBPM) [25]. The number of TFs that form at the larval stage determine the number of GSC niches at the adult stage [26–28], and are regulated by several signaling pathways that control cell division and timing of cell differentiation in the larval ovary, including the EcR [22], Hippo and Jak/Stat [27,28], Insulin [29] and Activin pathways [19]. Despite the recent advance in elucidating mechanisms that control the number of GSC niches and the temporal window in which they form [14], relatively little is known about the origin and specification of the somatic cell types of the GSC niche.

Notably, the origin and specification of cap cells, the main component of an active GSC niche is little understood. Cap cells (also called germarial tip cells) are first seen at the base of completed TFs at the transition from the 3rd larval instar to prepupal stage [16,17]. They appear to derive from the interstitial cells (also called intermingled cells) of the larval ovary that are maintained by Hh signaling from TFs [14, 30]. The formation of cap cells is accompanied by the establishment of GSCs [17]. The N pathway contributes to the development of cap cells [3]. A strongly increased number of functionally active cap cells per niche form in response to overexpression of the N ligand Delta (Dl) in germline or somatic cells, or the constitutive activation of N in somatic gonadal cells [8,22,31]. The ability of N to induce additional cap cells seems to depend on EcR signaling [22]. Loss of Dl or N in the germline had no effect on cap cells. However, loss of N in cap cell progenitors or Dl in TF cells caused a decrease in the number of cap cells [8,32]. A current model suggests that Dl signaling from basal-most TF cells to adjacent somatic cells together with Dl signaling between cap cells allows for a full complement of cap cells to form [8,32]. Furthermore, N protects cap cells from age-dependent loss as long as its activity is maintained by the Insulin receptor [32,33]. The Jak/Stat pathway, which operates downstream or in parallel to the N pathway in the niche [34], is not required for cap cell formation [34,35]. As cap cells were reduced in number but never completely missing when the N pathway components were compromised [8,31,32], the question remains whether N signaling is the only factor that is important for cap cell formation. Furthermore, no factor that operates downstream of N has been identified that is crucial for cap cell formation.

Here, we find that Traffic jam (Tj) is both required for cap cell specification and for the morphogenetic behavior of cap cells, enabling them to form a properly organized niche that can accommodate 2–3 GSCs. Tj is a large Maf transcription factor that belongs to the bZip protein family [36]. Its four mammalian homologs control differentiation of several cell types and are associated with various forms of cancer [37–39]. Tj is essential for normal ovary and testis development [36,40–42], and is only expressed in somatic cells of the gonad [36,43,44]. Interestingly, Tj is present in cap cells and escort cells but not in TFs [36]. We show that Tj is essential for the formation of the GSC niche. First, Tj regulates the behavior of cap cells, enabling them to form a cell cluster instead of a cell stack, which appears to be important for the formation of a normal-sized GSC niche with the capacity to support more than one GSC. Second, cap cells adopt the fate of TF cells in the absence of Tj function, and TF cells develop cap cell-like features when forced to express Tj, indicating that Tj specifies the cap cell fate. Genetic interactions suggest that Tj and N are required together for cap cell formation, but have different functions in this process. For somatic gonadal cells to adopt the cap cell fate, we propose that Tj has to be present to inhibit the TF cell fate and N has to be present to prevent the escort cell fate and/or produce the correct number of cap cell precursors.

## Results

### The structure of the female GSC niche in *Drosophila melanogaster*

To understand the defects in the stem cell niche of *tj* mutant ovaries, we reviewed the organization of the wild-type GSC niche, confirming and extending previous observations. The three somatic cell types of the GSC niche could be distinguished based on their position, cell and nuclear shape, and marker expression (Fig 1A–1D; S1 Table) [3,4,45]. The TF is a stack of disc-shaped cells (Fig 1A and 1B) [46]. The cap cell cluster at the tip of the germarium was either centered (Fig 1B) or formed an asymmetric streak that was attached to the base of a TF (Fig 1C) [6,47]. Cap cells had a rounded shape and were tightly packed in a cluster, with their nuclei in close proximity (Fig 1A–1D). Nuclei of escort cells had an angular (often triangular) appearance, and were bigger and more widely spaced than cap cell nuclei (Fig 1D). The anterior and posterior location of cap cells and escort cells, respectively, in relation to GSCs, produced a prominent gap between cap and escort cell nuclei (Fig 1A and 1D). GSCs made extensive contact to cap cells by forming a Bezel set-like rim of plasma membrane around each cap cell (Fig 1A and 1D–1F') [48]. We found that a GSC usually forms at least one prominent cellular protrusion toward cap cells, which distinguishes it from cystoblasts (Fig 1D, 1E' and 1F' arrowheads). These protrusions were seen with germline-specific markers that either label the cytoplasm (Vasa; Fig 1D) or the plasma membrane (*nos-Gal4 UAS-Gap43-mEos*; Fig 1E and 1F'). GSC protrusions were visible at various stages of the cell cycle as indicated by changes in the position of the spectrosome organelle (Fig 1E and 1F') [49]. The described morphological features helped identify cell types in the ovarian stem cell niche in addition to molecular markers.

Despite their different morphologies, cap cells have several markers in common with TF cells and some markers with escort cells (Fig 1B and 1D; S1 Table). Very few markers have been identified that seem to be specific for just one of these three cell types, but several markers showed differences in expression level (Fig 1B and 1D; S1 Table) [3,4,45]. Tj is expressed in cap cells and escort cells, which are located within the germarium and in contact to germline cells, but is not detected in TF cells, which form a stalk outside of the germarium (Fig 1B–1D) [36]. In addition, the cell that connects the cap cell cluster with the TF also contains Tj although at a considerably lower level than adjacent cap cells. We named this cell, which is disc-shaped similar to TF cells and aligned with TF cells, 'Transition cell' (Fig 1A and 1D). It might correspond to one of the basal cells of the TF that have been mentioned previously [47].

### Tj controls the arrangement of cells in the GSC niche

**Fig 2:**
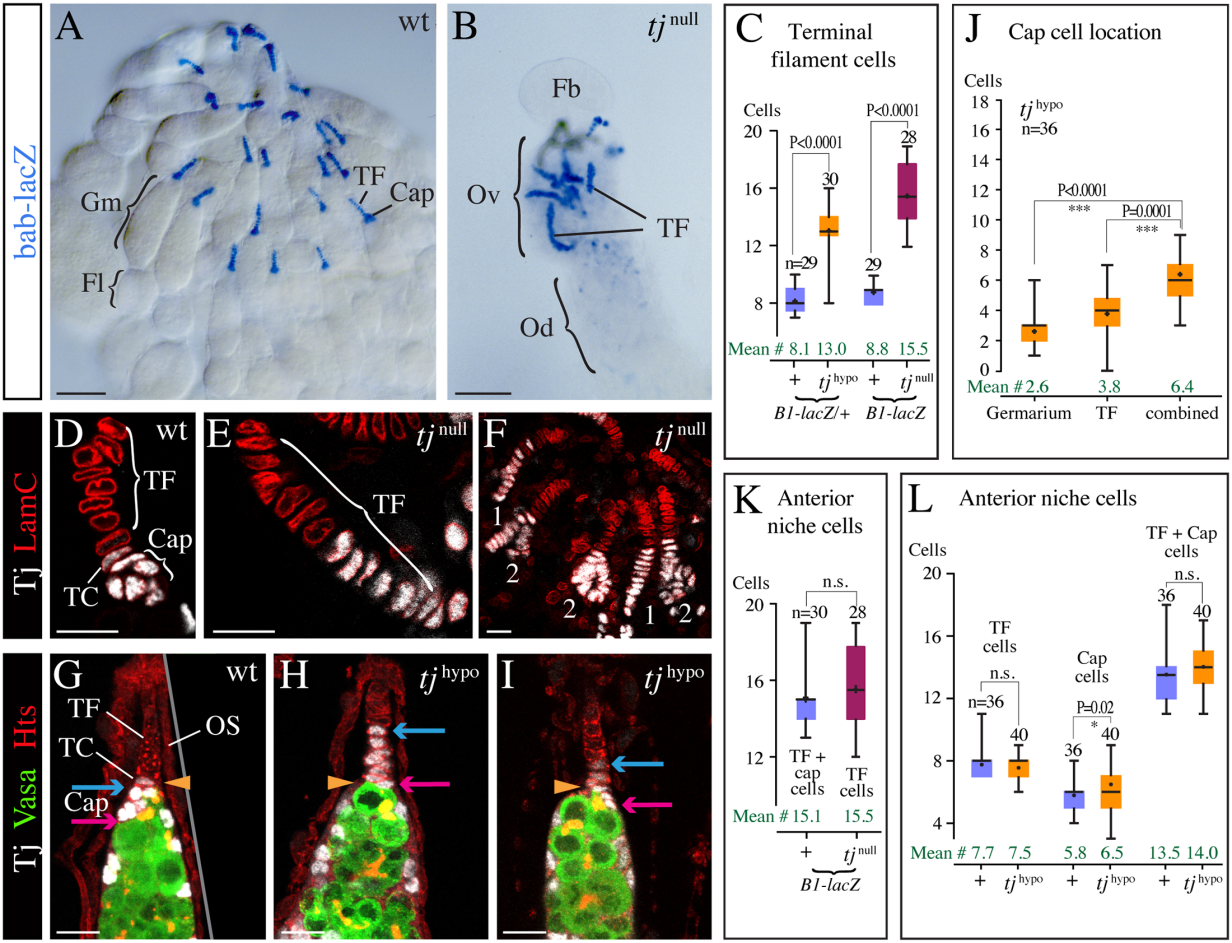
Loss of Tj affects the structure of the stem cell niche. (**A,B**) Adult ovaries. *bab-lacZ* highlights TFs and adjacent cap cells. (A) In a control (wt) ovary, a bundle of ovarioles is present, each with a TF at the tip, followed by a cap cell cluster (Cap), germarium (Gm) and series of follicles (Fl). (B) The rudimentary *tj*^*eo2*^/*tj*^*eo2*^ (*tj*^null^) mutant ovary (Ov) has irregularly distributed and abnormally long TFs, and germarium and follicles are missing. Od, oviduct; Fb, fatbody. (**C**) Comparison of the number of TF and cap cells in control (+), *tj*^*39*^*/tj*^*eo2*^ (*tj*^hypo^), and *tj*^*eo2*^*/tj*^*eo2*^ (*tj*^null^) ovarioles. (**D-F**) Niches of 2-day-old pupal ovaries. (D) In the control (wt), Tj-positive (white) cap cells are round and form a cluster at the base of the TF. TC, transition cell. (E,F) In a *tj*^*z4735*^*/tj*^*eo2*^ (*tj*^null^) ovary, cells expressing non-functional Tj protein are disc-shaped and either arranged in a single file, forming a stalk similar to the adjacent TF cells (E, F #1), or form a branched stalk (F #2). (**G-I**) Tip of an adult ovariole. Hts (red) outlines all cells, Vasa (green) marks germline cells, a yellow arrowhead marks the germarium/TF boundary, and red and blue arrows mark the posterior-and anterior-most cap cell, respectively. (G) In wild type (wt), all Tj-positive cells (white) are located inside the germarium, and the transition cell marks the boundary towards the TF. OS, ovariole sheath. (H,I) In a *tj*^*39*^*/tj*^*eo2*^ (*tj*^hypo^) ovary, a variable number of Tj-positive cells have become part of the TF (see anterior shift of the blue and red arrows). The Tj signal is weaker in the *tj*^hypo^ mutant (H,I) than in wild type (G). (**J**) Location of cap cells in *tj*^*39*^*/tj*^*eo2*^ (*tj*^hypo^) ovarioles. (**K,L**) Quantification of anterior niche cells, which include cap and TF cells, in *tj*^*eo2*^/*tj*^*eo2*^ (*tj*^null^) (K) and *tj*^*39*^*/tj*^*eo2*^ (*tj*^hypo^) ovarioles (L). Graphs (C,J-L) are 'box' (25–75 percentile) and 'whisker' (maxima/minima) diagrams, showing the median (bar) and mean (plus sign). *n*, sample size; n.s., not significant (P≥0.05). Genotypic markers: *bab-lacZ* (A,B), *1444-lacZ/+* (D-F), *B1-lacZ* or *B1-lacZ/+* (C,K). Anterior is up in all images. Scale bars: 50 μm in A,B; 10 μm in D-I.

In each ovariole of a wild-type ovary, a *bab-lacZ* positive TF and cap cell cluster are followed by a string of follicles (Fig 2A). Adult ovaries from *tj*^*eo2*^/*tj*^*eo2*^ null mutant females (*tj*^null^) lack germaria and follicles, and appear to mostly consist of TFs and ovariole sheath tissue (Fig 2B) [36,40]. Although TFs were seen properly oriented and enveloped by ovariole sheaths in some *tj* mutant ovaries, they were often not fully separated from each other, forming a tangled mass, or protruded from the ovary and adhered to extra-ovarian fat body tissue (Fig 2B).

Strikingly, the TFs appeared substantially longer in *tj*^null^ than in wild-type ovaries (Fig 2A and 2B). Instead of containing an average of 8 disc-shaped cells as in wild type ovaries (Fig 2C) [15], *tj*^null^ ovaries had TFs that contained on average 15 disc-shaped cells (Fig 2C). Moreover, cap cell clusters were not detected. To determine whether there is a connection between the larger stalks and the absence of cap cells, we used *tj*^*z4735*^, a genetic null allele that produces non-functional but detectable Tj protein to visualize cap cells [36]. The analysis of pupal *tj*^*z4735*^/*tj*^*eo2*^ ovaries showed that Tj-positive cells, which were never seen outside the germarium in wild type (Fig 2D), formed the basal portion of the TFs in mutant ovaries and were disc-shaped similar to normal TF cells (Fig 2E). The Tj-positive cells were often organized in a single file following the Tj-negative TF cells, although some stalks were found to branch or to form knob-like structures (Fig 2F). We conclude that cap cells form a TF-like stalk in the absence of *tj* function.

A similar niche defect was observed in a hypomorphic *tj* mutant. We isolated a very weak hypomorphic *tj* allele, *tj*^*39*^, through mobilization of *tj*-Gal4. It contains a P element fragment just upstream of the *tj* transcription unit and does not affect the *tj* coding region (S1A and S1B Fig; see Materials and methods). Although *tj*^*39*^ homozygous females had normally looking and functional ovaries, *tj*^*39*^ caused sub-fertility in trans to the *tj*^*eo2*^ null allele. *tj*^*39*^ produces full-length Tj protein, whereas *tj*^*eo2*^ produces a truncated isoform that is predicted to lack the DNA binding and leucine zipper domains due to a premature stop codon (S1C Fig) [36]. The amount of full-length Tj in *tj*^*39*^/*tj*^*eo2*^ ovaries was reduced to 40–50% of the wild-type value, whereas it was only reduced to approximately 70% in *tj*^*eo2*^/+ ovaries (S1C and S1C' Fig). Hypomorphic *tj*^*39*^/*tj*^*eo2*^ (*tj*^hypo^) ovaries had proper ovarioles with a germarium and developing follicles, but developed unusually pear-shaped germaria with age and had abnormal interfollicular stalks (see S3C and S3D Fig). Notably, *tj*^hypo^ ovaries displayed abnormally long TFs that included Tj-positive cells (Fig 2C and 2G–2I). In some cases, all Tj-positive cells anterior to GSCs were integrated into the TF (Fig 2H and 2J). More frequently, while most Tj-positive cells were part of the TF a few remained clustered at the tip of the germarium (Fig 2I and 2J), explaining the smaller cell number in TFs of *tj*^hypo^ compared with *tj*^null^ mutant ovaries (Fig 2C). Moreover, stalk-forming Tj-positive cells were often disc-shaped and arranged in a single row similar to normal TF cells, and even clustered Tj-positive cells often appeared flatter in shape than regular cap cells (Fig 2H and 2I). The range in cellular behavior suggests that these Tj-positive cells have a hybrid character, having gained TF cell characteristics and lost cap cell features to a variable degree. The hypomorphic *tj* mutant phenotype supports the notion that Tj is important for niche organization, enabling cap cells to form a cluster inside the germarium where they can contact GSCs.

### Tj is required for the specification of cap cells

If additional TF cells form at the expense of cap cells, as our data suggest, one would expect the number of cells in the TF of *tj*^null^ ovaries to equal the sum of TF cells and cap cells in wild-type ovaries. Indeed, those numbers were similar when we counted the cells of individual stalks using the markers *B1-lacZ* and Lamin C (LamC) that both label TF and cap cells but not escort cells (Fig 2K, S1 Table). In *tj*^hypo^ ovaries, a combination of the markers LamC, labeling TF and cap cells, and Tj, labeling cap and escort cells, allowed us to clearly distinguish all three cell types. The number of cap cells in our controls was similar to previous reports (Fig 2L) [6,8,32]. A minor increase in the number of cap cells in *tj*^hypo^ mutant ovaries was observed in two out of three genetic backgrounds, with an average of 6.5–8.3 cap cells in *tj*^hypo^ mutants compared with 5.8–7 cap cells in controls (Fig 2L). However, there was no significant difference in the number of TF cells or in the combined number of TF and cap cells between control and *tj*^hypo^ ovarioles (Fig 2L). The total number of stalk-forming cells was lower than the combined count of TF and cap cells in *tj*^hypo^ ovarioles, which was expected as not all cap cells become part of the stalk in *tj*^hypo^ ovaries (Fig 2J). Taken together, our quantitative analysis indicates that the number of anterior niche cells remained unaffected in *tj* mutant ovaries, suggesting that Tj regulates the fate of niche cells but not their numbers.

**Fig 3:**
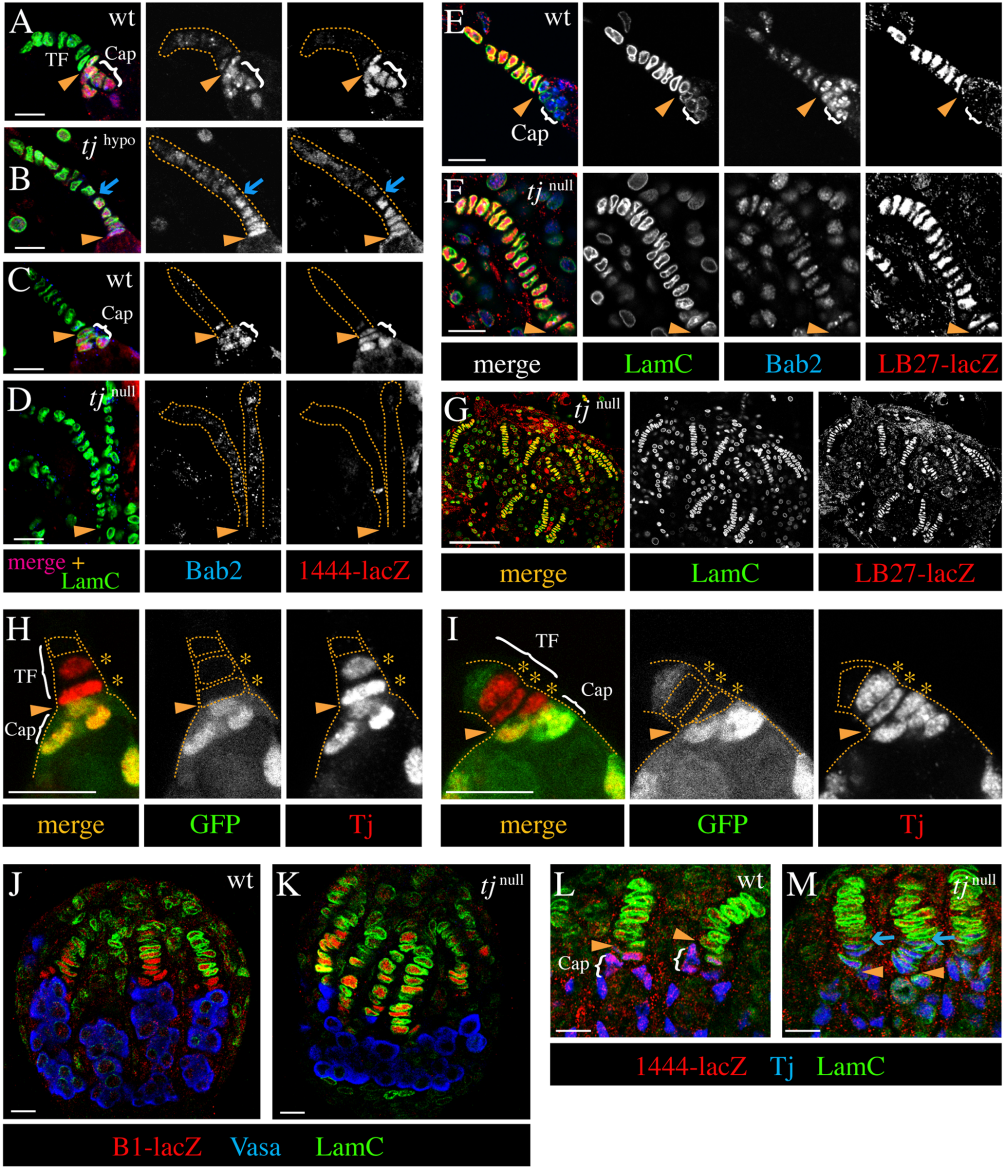
Tj controls the morphogenetic behavior and specification of cap cells. Yellow arrowheads mark the germarium/TF boundary. (**A-G**) GSC niches of adult ovaries. LamC identifies TF and cap cells. In the control (wt) (A,C,E), cap cells form a cluster inside the germarium and TF cells form a stalk outside the germarium. In ovaries of *tj*^*39*^/*tj*^*eo2*^ (*tj*^hypo^) (B) and *tj*^null^ mutants (*tj*^*eo2*^*/tj*^*eo2*^ in D, *tj*^*eo2*^*/tj*^*Df1*^ in F,G), the cluster is missing and the stalk is abnormally long. (B) In a *tj*^hypo^ ovary, the basal cells of the elongated stalk (between arrow and arrowhead) express markers in a cap cell-specific manner (strong Bab2 and *1444-lacZ* signals), in contrast to distal cells in the stalk. (D,F,G) In *tj*^null^ ovaries, all cells in the elongated stalks express markers similar to normal TFs (weak and strong expression of Bab2 (D,F) and *LB27-lacZ* (F,G), respectively, and no detection of *1444-lacZ* (D). (**H,I**) Adult GSC niches with *tj*^*z4735*^ mutant cell clones. *tj*^*z4735*^ mutant cap cells, which only express the non-functional isoform of Tj (red) and lack GFP (green), are located in the TF (above the arrowhead) and display the flat shape of TF cells (asterisks), whereas control cap cells, which co-express Tj and GFP, are located in the germarium (below the arrowhead). (**J-M**) Prepupal ovaries. TFs are longer in *tj*^null^ ovaries (*tj*^*eo2*^*/tj*^*eo2*^ in K, *tj*^*z4735*^*/tj*^*eo2*^ in M) than in control ovaries (J,L). (J,K) *B1-lacZ* (red) labels cap and TF cells. LamC (green), which is only seen in the distal half of TFs in the control ovary, is present in all *B1-lacZ*-positive cells of the *tj*^null^ ovary. The germ cell population, marked by Vasa (blue), is not divided into ovarioles in the *tj*^null^ ovary in contrast to the control. (L,M) *1444-lacZ* (red), which labels nascent cap cells in the control ovary is not detected in the *tj*^null^ ovary. Tj (blue) is seen in cap cells but not in TFs in the control ovary. In the *tj*^null^ ovary, which expresses a mutant Tj isoform, Tj is seen in the basal portion of the elongated TFs (between arrowhead and arrow). Genotypic markers: *1444-lacZ/+* (A,B,L,M) or *1444-lacZ* (C,D), *LB27-lacZ/+* (E-G), *Ubi-GFP* (H,I), and *B1-lacZ* (J,K). Anterior is up. Scale bars: 10 μm in A-F,H-M; 50 μm in G.

Cap cells adopted the shape and morphogenetic behavior of TF cells in *tj* mutants. To determine whether a reduction of Tj causes indeed a change in cell fate, we used several markers that differ in their expression in the two cell types (S1 Table). In *tj*^hypo^ ovaries, cells in the upper portion of the terminal stalks expressed low levels of Bab2 and background levels of *1444-lacZ* similar to wild-type TF cells (Fig 3A and 3B). In contrast, the lower portion of the *tj*^hypo^ mutant stalks expressed high levels of Bab2 and *1444-lacZ* (Fig 3B), which is typical of wild-type cap cells (Fig 3A). Furthermore, the markers LamC and *B1-lacZ*, which stained TF cells more intensely than cap cells in wild type (S2A Fig), showed a stronger signal in the upper than in the lower portion of *tj*^hypo^ mutant stalks (S2B Fig). This indicates that the additional stalk cells retain the expression profile of cap cells despite the dramatic change in morphology. However, *LB27-lacZ*, a TF-specific marker that is expressed in a complementary pattern to Tj in wild type (S2E Fig), was sometimes seen at reduced levels in the stalk-forming Tj-positive cap cells in *tj*^hypo^ ovarioles, (S2F Fig), pointing toward a potential defect in cell specification.

To further investigate the function of Tj in cap cell specification, we evaluated the expression of markers in *tj*^null^ ovaries, using different allelic combinations, including *tj*^*eo2*^, *tj*^*z4735*^, and a newly generated transcriptional null mutation, *tj*^*Df1*^ (see Materials and methods, S1A Fig). The absence of the cap cell-specific marker *1444-lacZ* (Fig 3C and 3D), the weak signal of Bab2 (Fig 3C–3F), the strong signals of LamC (Fig 3E–3G and S2C and S2D Fig) and *B1-lacZ* (S2C and S2D Fig), and in particular the presence of the TF-specific marker *LB27-lacZ* (Fig 3E–3G and S2E and S2G Fig) throughout the elongated stalk of *tj*^null^ mutants are all indicative of a shift in cell fate. This expression profile is consistent with the TF cell-like disc-shaped morphology and stalk-forming behavior, and we therefore conclude that additional TF cells form at the expense of cap cells in the absence of Tj function.

To test whether the effect of Tj depletion on the fate of cap cells is cell-autonomous, we induced *tj*^*null*^ mutant cell clones in the GSC niche during the larval stage. We focused on germaria that contained mutant anterior niche cells (cap and/or TF cells) but did not contain mutant escort cells close to cap cells to separate the *tj* loss-of-function effect on cap cells from that on escort cells (S2H and S2I Fig). Use of the *tj*^*z4735*^ allele allowed us to distinguish between regular TF cells and transformed cap cells, as the latter expressed Tj. In cases of mosaic cap cell groups, *tj* homozygous mutant cells usually looked like TF cells and had become part of the TF, whereas the non-mutant cap cells were rounded and clustered posterior to the mutant cells in the germarium (Fig 3H and 3I and S2H and S2I Fig). The abnormal behavior of *tj* mutant cap cells was independent of whether the neighboring bona fide TF cell was a *tj* mutant cell (GFP negative, Fig 3H) or a control cell (GFP positive; Fig 3I). Our clonal analysis shows that Tj is cell-autonomously required for cap cell morphology and behavior.

To determine whether presumptive cap cells are abnormally specified at the time of their origin or are not able to maintain the cap cell fate when Tj is depleted, we looked at developing ovaries at the stage of cap cell formation. Cap cells develop gradually during the late 3rd instar larval and early prepupal stage following the formation of TFs [17]. Already at the prepupal stage, the TFs were longer in *tj*^null^ ovaries than in wild-type ovaries, consisting of an increased number of cells that were aligned in a single file (Fig 3J and 3K). The expression of niche markers at the prepupal stage is different from the adult stage (S2 Table). All cells of the mutant stalks showed prominent LamC staining in contrast to control prepupal ovaries, where this marker was not detected in cap cells and only weakly expressed in basal TF cells (Fig 3J–3M; S2 Table). Moreover, *1444-lacZ*, which was co-expressed with Tj in cap cells of prepupal wild-type ovaries (although not yet seen in escort cells), was not detected in the Tj-positive stalk cells of *tj*^null^ ovaries (Fig 3L and 3M). This shows that the defects in cap cell specification already develop at the time of niche formation. Taken together, our data indicate that all the anterior niche cells adopt a TF cell fate in the absence of Tj function, implicating Tj as a crucial factor for cap cell specification.

### TF cells that are induced to express Tj adopt cap cell-like characteristics

**Fig 4:**
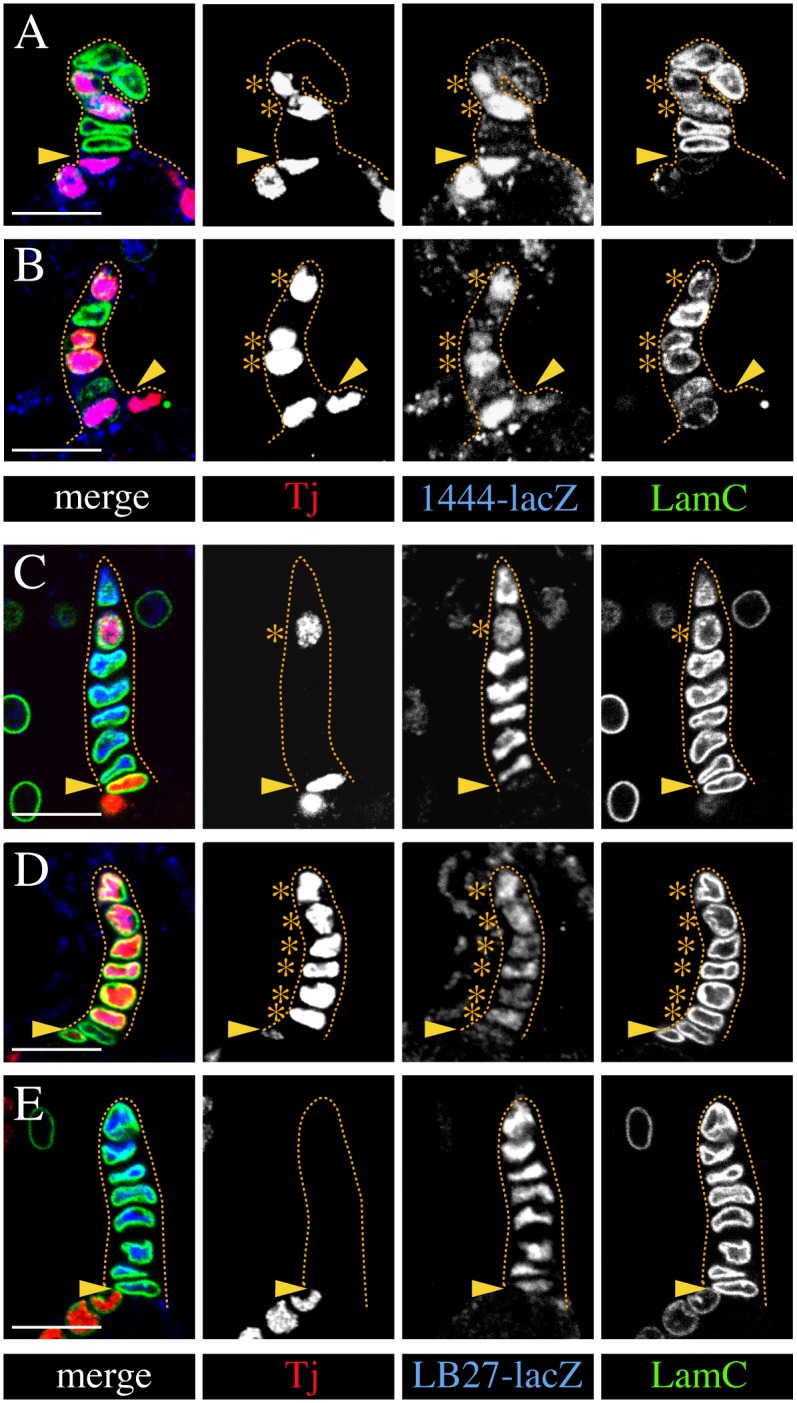
Ectopic expression of Tj in TF cells induces cap cell-like characteristics. All images show a single TF. Tj-expressing TF cells were induced in the larval ovary before TFs form and are marked by an asterisk. An arrowhead marks the TF/germarium boundary. (**A,B**) In mosaic TFs, Tj-positive cells strongly express the cap cell marker *1444-lacZ* in contrast to adjacent control cells. Also, the LamC signal appears weaker in Tj-positive cells than in normal Tj-negative TF cells. (**C-E**) Expression of the TF cell-specific marker *LB27-lacZ* is substantially reduced in Tj-positive cells (C,D) when compared to control TF cells (C,E). Note the rounded shape of Tj-expressing TF cells when compared to the flat shape of control TF cells in mosaic stalks (A-C). Genotypic markers: *1444-lacZ/+* (A,B), *LB27-lacZ/+* (C-E). Anterior is up in all panels. Scale bars: 10 μm.

As Tj is required for cap cell specification, we asked whether expression of Tj in anterior niche cells would be sufficient to induce the cap cell fate. We induced Tj-expressing cells in TFs in early 3rd instar, before TFs begin to form by cell intercalation [15,16], and analyzed mosaic TFs in adult ovaries. The frequency of mosaic TFs was similar in ovaries with clonal expression of either Tj or GFP, suggesting that Tj expression did not affect the survival of TF cells (S3 Table). Strikingly, Tj-positive TF cells expressed high levels of *1444-lacZ* and low levels of LamC similar to wild-type cap cells (Fig 4A and 4B). In addition, the expression of *LB27-lacZ* was strongly reduced in Tj-positive TF cells compared with control TF cells, although not completely abolished (Fig 4C–4E). Thus, expression of Tj in TFs resulted in ectopic expression of a cap cell marker and partial suppression of a TF cell marker.

Despite the changes in marker expression, Tj-expressing TF cells remained in the TF (Fig 4A–4C), and even formed a stack of aligned cells when all TF cells expressed Tj (Fig 4D and 4E). However, Tj-expressing TF cells appeared rounder than their control neighbours (Fig 4B and 4C). Cell shape analysis confirmed that Tj-expressing TF cells have a significantly increased height and decreased width compared to control TF cells (Table 1), which is consistent with a rounder, more cap cell-like morphology. Our data indicate that Tj induces TF cells to adopt molecular and morphological characteristics of cap cells.

**Table 1:** Ectopic expression of Tj affects the shape of TF cells.

Transgene	Number of TF cells measured (*n*)	Width Mean [μm]	Height Mean [μm]	Height/Width Ratio	
				Mean (s.d.)	*P* value
***UAS-GFP***	114	5.57	2.58	0.48 (0.14)	<0.0001
***UAS-tj*** ***UAS-GFP***	99	5.08	3.09	0.62 (0.14)	

Clonal expression of *Act5C-Gal4* was used to drive expression of *UAS-tj*^*1(2)*^ in combination with *UAS-GFP* in TF cells. *UAS-GFP* alone was used as a control.

### The stalk-like organization of cap cells in ovaries with low Tj expression causes a reduction in the number of GSCs

**Fig 5:**
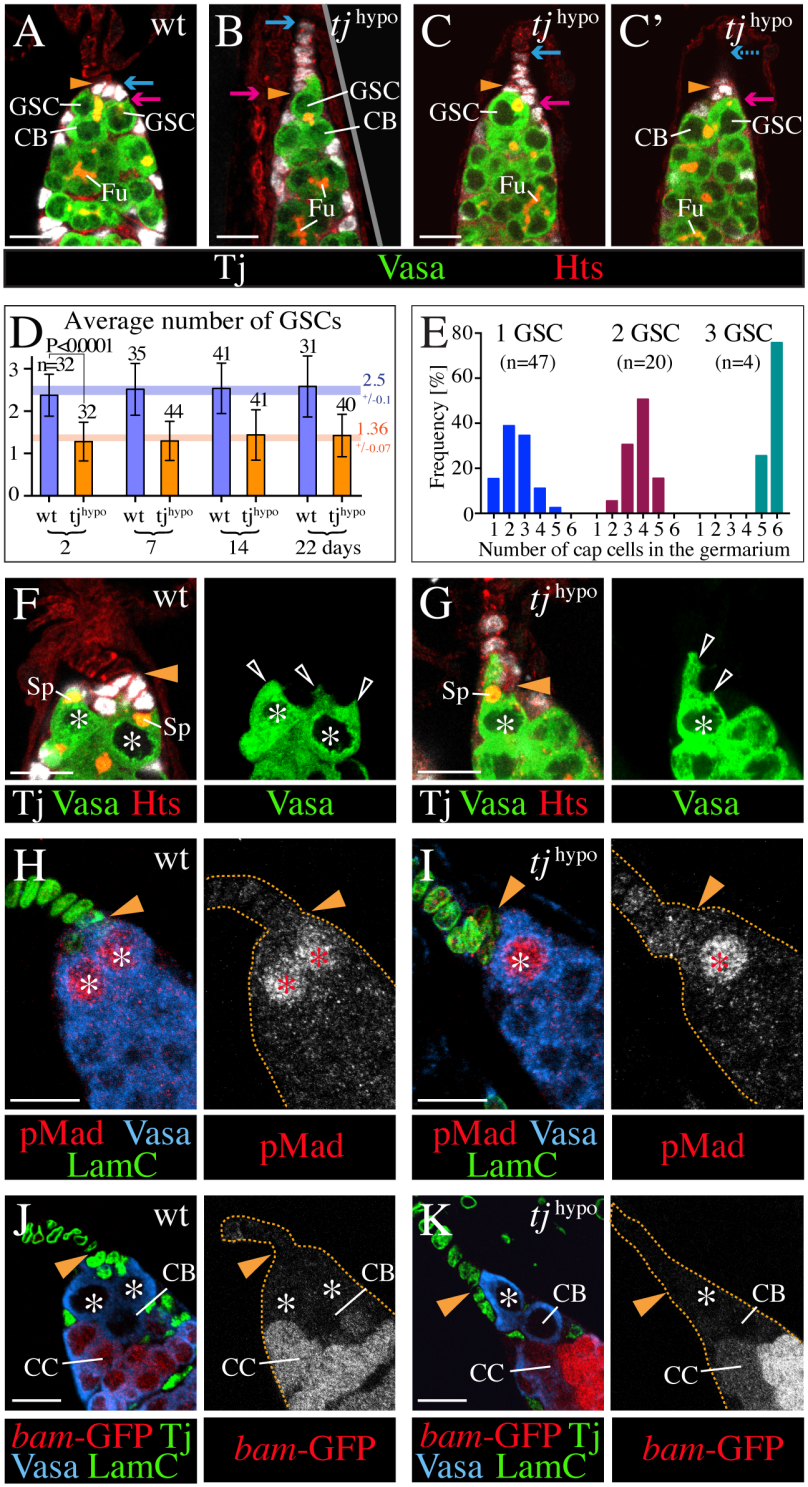
Partial reduction of Tj affects the number but not maintenance of GSCs. Images of the GSC niche in *tj*^*39*^*/tj*^*eo2*^ mutant (*tj*^hypo^) and control (wt) germaria. Vasa labels germline cells, Tj labels cap cells, and LamC labels TF and cap cells. Hts highlights the spectrosome (Sp) of GSCs and pre-cystoblasts/cystoblasts (CB), and the fusome (Fu) of germline cysts, and outlines all cells. A yellow arrowhead marks the TF/germarium boundary. Red and blue arrows mark the posterior-and anterior-most cap cell, respectively (A-C'), and asterisks mark GSCs (F-K). (**A-C'**) In contrast to a wild-type niche (A) a *tj*^hypo^ niche often contains only a single GSC (B), but sometimes has two GSCs (C,C' are two sections of the same germarium). A dividing GSC that produces a cystoblast is seen in panels A and C'. (**D**) The graph compares the number of GSCs in control (*bam-GFP*) and *tj*^hypo^ germaria (*tj*^*39*^*/tj*^*eo2*^; *bam-GFP)* from females aged for the indicated time interval. Mean + s.d. are indicated. *n*, sample size. The difference in GSC numbers between the two genotypes is significant for each time point (P<0.0001; Student T-test, unpaired, two-tailed, equal variance). Within each genotype, differences in GSC numbers between time points were not significant (P>0.05; ANOVA one-way). For each genotype, a colored number and horizontal bar indicate the mean and differences in the mean between time points. (**E**) The graph shows the relationship between the number of GSCs and the number of cap cells that were located in the germarium of *tj*^hypo^ ovarioles. Among the analyzed ovarioles (n = 72), germaria with 3 GSCs were rare. (**F,G**) Protrusions of GSCs (open arrowheads) partially wrap the cap cells (white) in a wild-type germarium (F). The single GSC of a *tj*^hypo^ germarium has an unusually long protrusion that contacts ectopically positioned cap cells outside the germarium (G). In both genotypes, GSCs have anteriorly located spectrosomes. (**H-K**) Similar to GSCs in the control (H,J), the anterior-most germline cell in the *tj*^hypo^ germarium shows nuclear pMad (I), and lacks *bam*-GFP (K). *bam*-GFP is prominent in dividing cystocytes (CC) in both genotypes (J,K). Anterior is up in all images. Scale bars: 10 μm in A-C, F-K.

The spread-out cluster of cap cells in a wild-type germarium provides a large contact surface for anchorage of GSCs [25]. In *tj*^hypo^ ovarioles, however, most cap cells are recruited into the TF, and few remain in the germarium, potentially limiting their availability to GSCs. In cases where all cap cells form a single file stalk, only the basal-most cap cell would offer a physical GSC anchor point. Therefore, we asked whether the abnormal organization of cap cells in *tj*^hypo^ ovarioles might affect the number or maintenance of GSCs. We noticed that the germaria of *tj*^hypo^ ovaries were often unusually narrow (Fig 5A and 5B), harbouring only 1–2 GSCs (Fig 5B, 5C' and 5D) in contrast to 2–3 GSCs in wild-type germaria (Fig 5A and 5D) [6,50]. As Tj was found to be neither expressed nor required in the germline [36], the reduction in GSC numbers is likely caused by the observed defects in the stem cell niche of *tj* mutants.

Only a single GSC was present when all cap cells had joined the TF in *tj*^hypo^ ovarioles (Fig 5B). The number of GSCs per mutant ovariole increased with the number of cap cells that remained in the germarium (Fig 5C, 5C' and 5E). The majority of ovarioles with 2–3 cap cells within the germarium had still only one GSC, whereas those with four and six cap cells in the germarium had usually two and three GSCs, respectively (Fig 5E). Therefore, the number of GSCs in *tj*^hypo^ mutant ovarioles correlates with the number of cap cells that remain in the germarium instead of the total number of cap cells.

These data are consistent with an approximate 2:1 ratio of cap cells to GSCs that has been observed for wild-type ovarioles [6,8], the finding that GSCs require direct contact to cap cells [7,8,22,51], and the observation that GSCs partially envelop more than one cap cell with cytoplasmic extensions in wild type (Fig 5F). If, however, a contact to more than one cap cell is required to support a GSC, how could any GSC exist if all cap cells are arranged in a stalk. Interestingly, we discovered that in 79% of those cases (n = 14), the GSC produced a long cellular protrusion that reached far into the TF of a *tj*^hypo^ ovariole, allowing it to contact at least two cap cells (Fig 5B and 5G). In comparison, when two or more than two cap cells remained in the germarium, the frequency of unusually long GSC protrusions was only 42% (n = 12) and 0% (n = 11), respectively.

To determine whether the cells that we identified as GSCs based on morphological criteria are bona-fide GSCs, we analyzed the activity of the Dpp signaling pathway by probing for the presence of phosphorylated Mothers against dpp (pMad), the effector of this pathway [52]. In wild-type germaria, nuclear pMad identifies GSCs (Fig 5H) [11]. Similarly, in *tj*^hypo^ germaria, nuclear pMad was restricted to germline cells that abutted cap cells and had the morphology of GSCs (Fig 5I). In most *tj*^hypo^ ovarioles, only a single germline cell was positive for pMad, consistent with a reduced number of GSCs. The staining intensity of nuclear pMad was comparable between GSCs of wild-type (n = 23) and mutant germaria (n = 25). Consistent with this finding, *bam* expression, which is repressed by pMad to prevent differentiation of GSCs [10,11], was not detected in GSCs but was seen in differentiating germline cysts in both *tj*^hypo^ and wild-type germaria (Fig 5J and 5K). Together, this suggests that Dpp signaling from niche cells is active and confirms the presence of GSCs in the *tj*^hypo^ mutant.

An aging experiment that assessed whether reduced expression of Tj affects the maintenance of GSCs showed that the number of GSCs remained stable over a period of three weeks in wild-type and *tj*^hypo^ mutant ovarioles (Fig 5D). Presence of germaria and rows of follicles of successive developmental stages in 2–22 day-old *tj*^hypo^ ovaries similar to wild-type ovarioles confirms the proper maintenance of GSCs (S3A–S3D Fig). Absence of *bam-*GFP expression (S3E and S3F Fig) and presence of nuclear pMad in the anterior-most germline cells that contact cap cells (S3G and S3H Fig) are consistent with the conclusion that GSCs, although smaller in number, are maintained normally in *tj*^hypo^ mutant ovaries.

### Lack of Tj function disrupts GSC establishment and causes loss of germline cells

**Fig 6:**
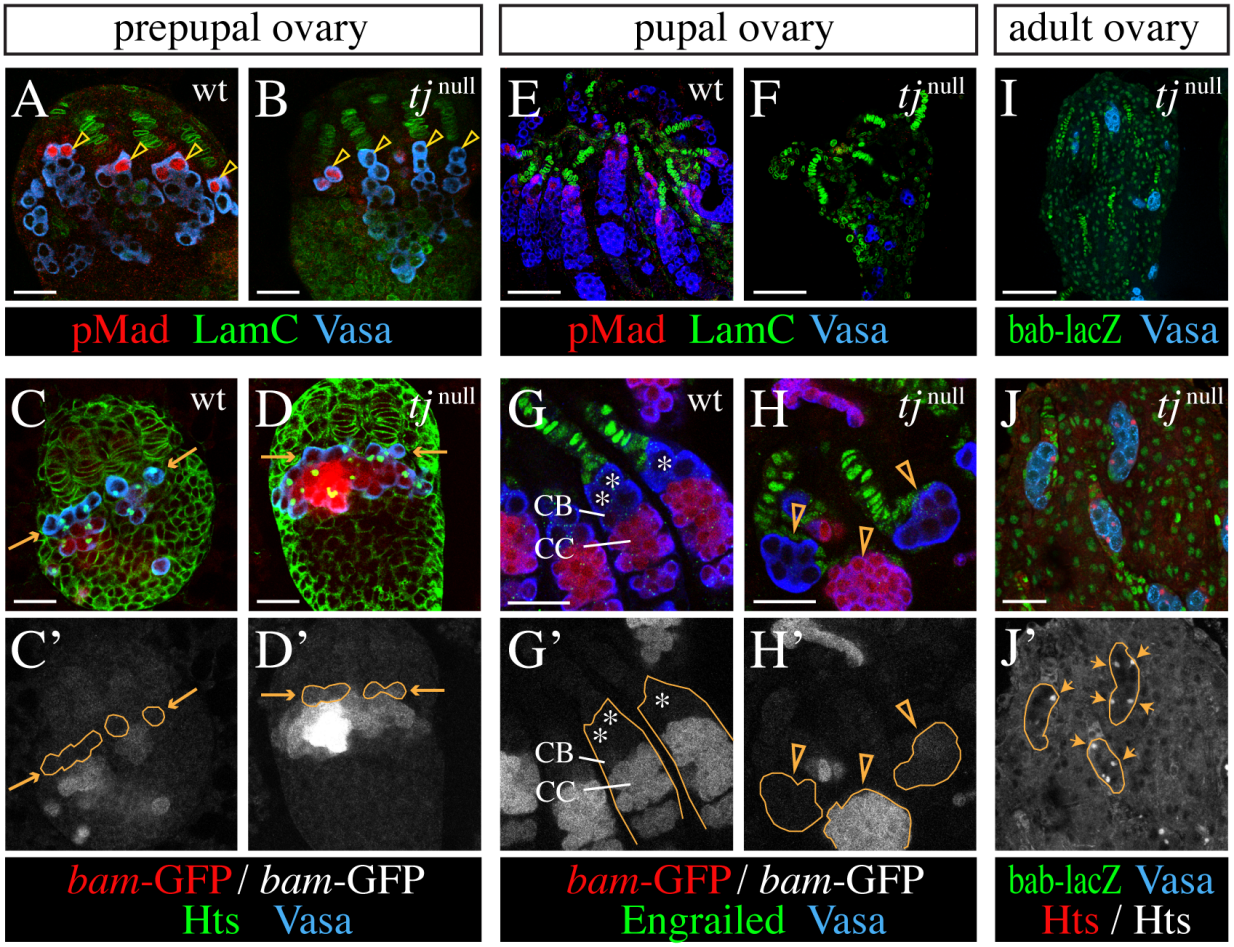
Loss of Tj function impairs GSC formation and causes loss of germline cells. Images compare the germline phenotype of *tj*^*eo2*^*/tj*^*eo2*^ mutants (*tj*^null^) with a control (wt) at different stages of gonad development. Vasa (blue) marks germline cells. LamC (A,B,E,F), Engrailed (G,H), or *bab-lacZ* (I,J) highlight TFs in green. Hts (green in C,D) marks spectrosomes and fusomes and outlines all cells. (**A-D'**) In a wild-type ovary at the prepupal stage, GSCs (arrowheads) occupy the anterior-most row in the germ cell population as indicated by the presence of pMad (red) (A) and the absence of *bam*-GFP (red in C and white in C'). Of the anterior-most row of germline cells in a *tj*^null^ ovary, only a few are positive for pMad (arrowheads) (B), and some express *bam*-GFF (D,D'). The anterior-most row of the germ cell population is marked by arrows and/or a stippled line in C-D'. (**E-H'**) At the mid-pupal stage, wild-type ovarioles are already filled with developing germ cells and contain pMad-positive GSCs next to TFs (E), whereas few germline cells remain in a *tj*^null^ ovary and pMad is not detected (F). (G,G') In wild-type ovarioles, *bam*-GFP is not detected in GSCs (asterisk), barely visible in adjacent cystoblasts (CB) and strongly expressed in cystocytes (CC). (H,H') The few persisting germline clusters in a *tj*^null^ ovary that are associated with TFs show different levels of *bam*-GFP expression (arrowheads). (**I-J'**) Remaining germ cells in adult *tj* mutant ovaries are organized in small clusters and are usually associated with TFs (I,J), and contain a spectrosome (arrows in J') that is marked by Hts (red in J and white in J'). Genotypic markers: *bam-GFP* (C-D',G-H'), *bab-lacZ/+* (I-J'). Anterior is up in all panels. Scale bars: 20 μm in A-D,G,H,J; 50 μm in E,F,I.

As cap cells are considered essential for GSC establishment and maintenance [3,4], and our analysis indicates that cap cell specification depends on Tj, we expected a loss of GSCs in *tj*^null^ mutant ovaries. In ovaries of wild-type prepupae, the anterior-most row of germline cells next to the newly formed niches represented GSCs as indicated by the presence of nuclear pMad (Fig 6A) [11,17]. In *tj*^null^ prepupal ovaries (n = 15), 68% of the TFs were not associated with any germline cells (orphan TFs). The remaining TFs captured usually no more than one germline cell. Surprisingly, we found that some of the TF-associated germline cells displayed nuclear pMad (Fig 6B), although their number was very low, with a mean of 2.3 nuclear pMad-positive cells in a *tj*^null^ ovary (n = 17) compared with 13.6 in a wild-type prepupal ovary (n = 11). As orphan TFs in *tj* mutant ovaries might potentially result from the abnormal distribution of germ cells and interstitial cells [36], we asked whether the number of pMad-positive cells per occupied TFs was different from wild type. Taking into account that even in wild-type prepupal ovaries only a third of niche-associated germline cells were positive for nuclear pMad, that only 32% of the mutant TFs were occupied by a germline cell, and that the mean number of TFs was reduced by 20% in *tj*^null^ ovaries (15 and 19 TFs in *tj*^null^ (n = 18) and wild-type ovaries (n = 17), respectively), we calculated a 33% reduction of nuclear pMad-positive germline cells per occupied TF in mutant ovaries (0.48 and 0.72 nuclear pMad-positive cells per occupied TF in *tj*^null^ and wild-type ovaries, respectively). Notably, *bam*-GFP, which was absent in GSCs of wild-type ovaries was detected in some of the TF-associated germline cells in *tj*^null^ ovaries (Fig 6C and 6D'), suggesting entrance into differentiation [11,17].

To follow the fate of germline cells in *tj*^null^ mutant ovaries, we analyzed ovaries at the pupal and adult stage. We had previously reported that more than 25% of *tj*^null^ ovaries from young adult females were devoid of germline cells [36]. Already at the mid pupal stage, when the germaria had matured in wild-type ovaries, and displayed a largely expanded germline cell population (Fig 6E), *tj*^null^ ovaries contained only a few small and scattered germline cells or cell clusters and pMad staining was drastically reduced (Fig 6F), suggesting a rapid loss of germline cells during the pupal period. The *bam*-GFP signal that revealed early germline cysts in control ovarioles (Fig 6G and 6G') ranged from non-detectable to prominent in the remaining germline clusters in *tj*^null^ ovaries (Fig 6H and 6H'). In adult ovaries, where 96% of the few remaining germline cell clusters (n = 45; 10 ovaries) were associated with TFs (Fig 6I and 6J), pMad-positive cells were rare, and only present in 9.5% of the clusters (n = 21; 18 ovaries). Some clusters consisted of individual germline cells, as indicated by spectrosomes (Fig 6J and 6J'), others had undergone transit amplification with incomplete cytokinesis, displaying branched fusomes [36]. Taken together, we infer that the complete loss of Tj activity severely compromises GSC establishment and maintenance.

### The germline influences the number but not the organization of cap cells

**Fig 7:**
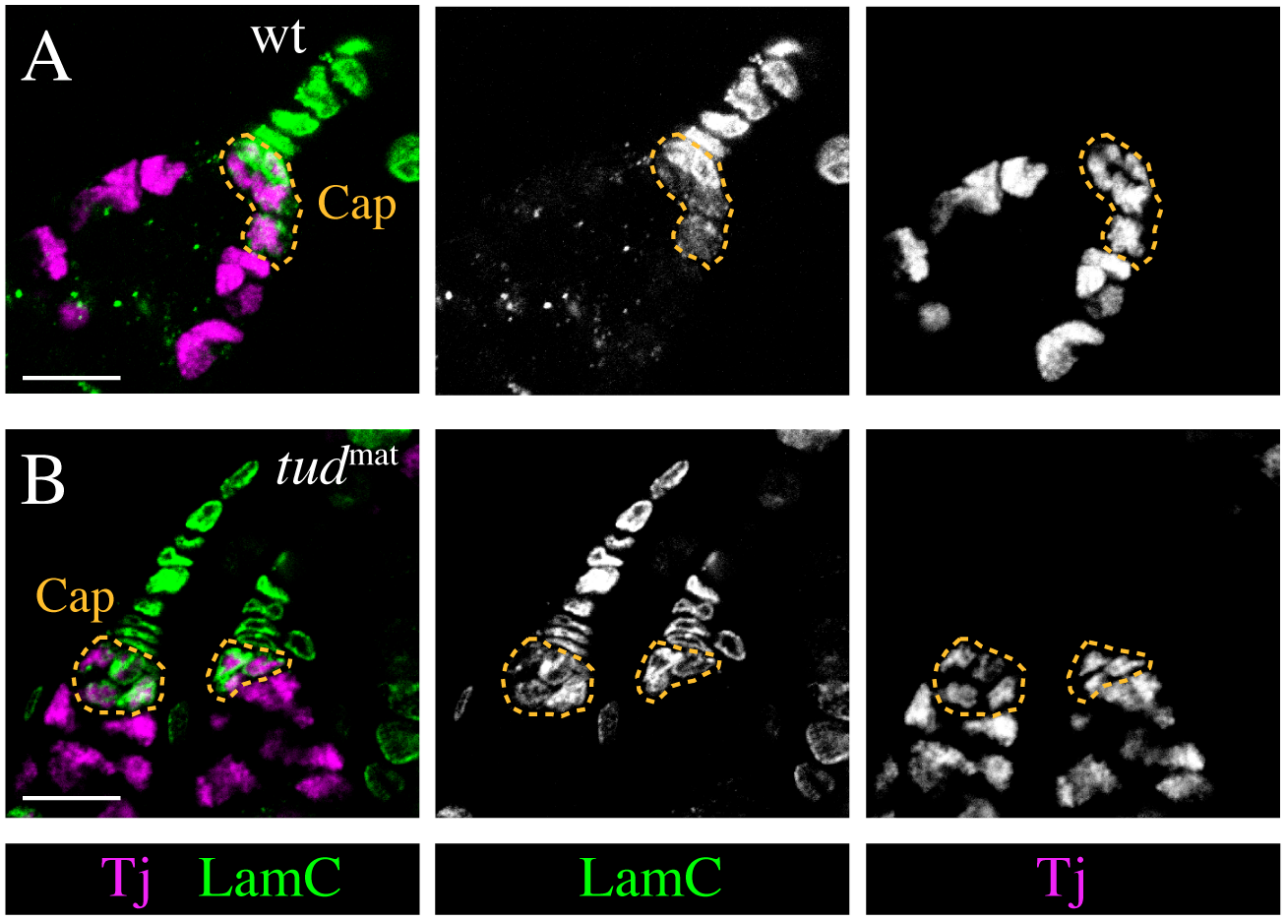
Cap cells form a cluster in the germarium in the absence of germline cells. Images show the GSC niche. TF cells display LamC (green), escort cells express Tj (magenta), and the cap cells, which are highlighted by a stippled line, are positive for both markers. Cap cells form a cluster posterior to the stack of TF cells and are part of the germarium in ovarioles of a *tud*^*1*^*/tud*^*b45*^ maternal effect mutant ovary that lacks the germline (B) similar to wild type (wt) (A). Anterior is up. Scale bars: 10 μm.

To determine whether the reduction/loss of germline cells in *tj* mutant ovaries is responsible for the recruitment of cap cells into the TF we analyzed the behavior of cap cells in *tudor* and *oskar* maternal effect mutants (*tud*^mat^ and *osk*^mat^, respectively) that lack germline cells [53,54]. The number of cap cells was reduced in *tud*^mat^ ovaries (average of 3.7 cap cells; n = 49) compared with wild type (average of 7.7 cap cells; n = 11; p<0.0001). This indicates that cap cells can form in the absence of a germline, although their number is reduced, which is consistent with previous reports [8,51,55]. Importantly, however, the number of TF cells was not increased but rather slightly reduced in *tud*^mat^ ovaries (average of 7.1 TF cells; n = 44) compared with wild type (average of 8 TF cells; n = 11; p<0.01), indicating that the reduced number of cap cells is not caused by a change from cap cell to TF cell fate. Furthermore, the remaining cap cells had a rounded shape, were organized into a cluster that resided within the germarium, and expressed Tj similar to wild-type cap cells (Fig 7A and 7B). Similarly, cap cells were organized as a cluster in *osk*^mat^ mutant ovaries. These findings indicate that the number but not the morphology or spatial arrangement of cap cells depends on the presence of GSCs.

### Tj and the N pathway cooperate in the formation of cap cells

**Fig 8:**
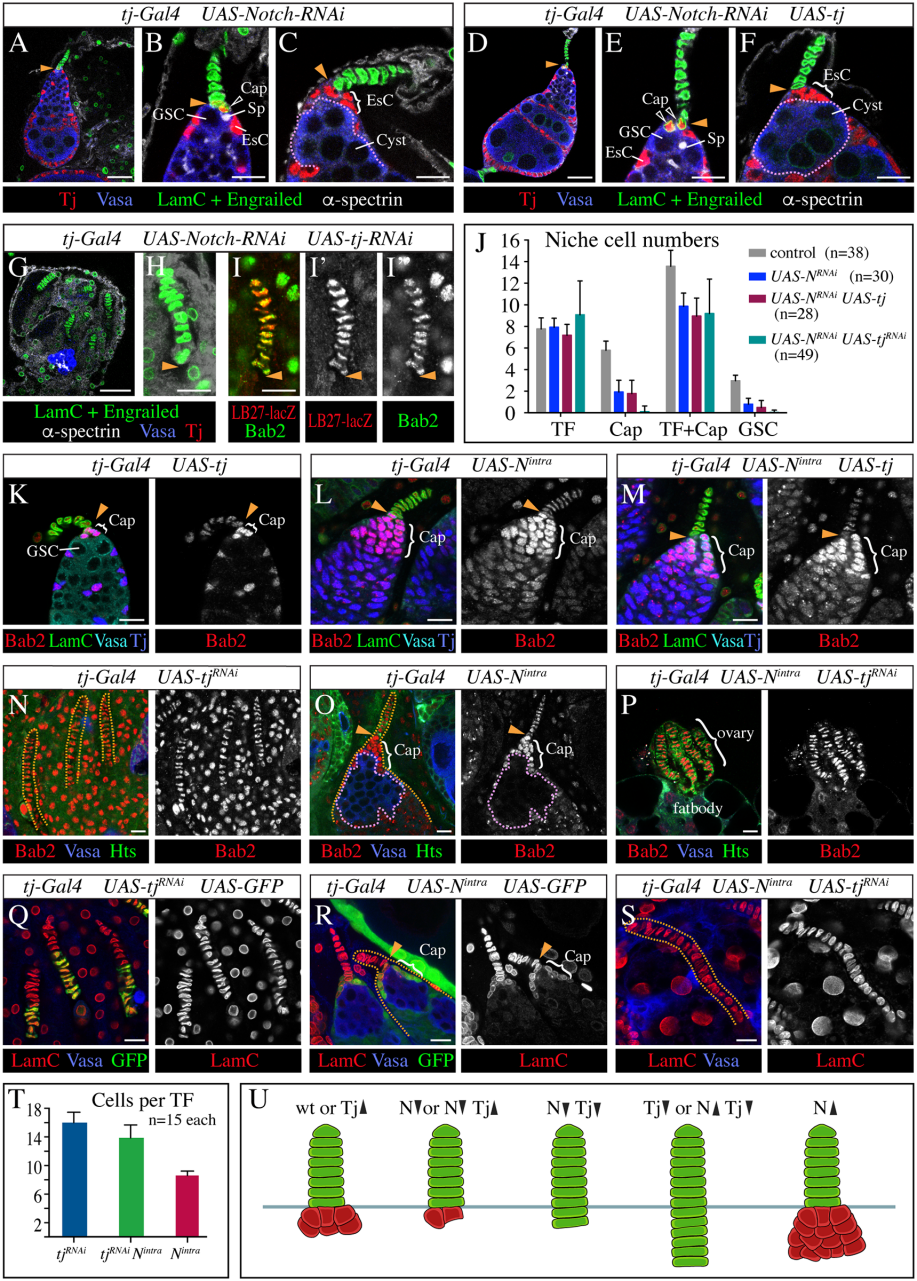
Tj and N have different effects on cap cell development. A yellow arrowhead marks the posterior end of the TF. **(A-J)** The effects on cap cells caused by N depletion and altered Tj expression. (A-C) *N* knockdown by RNA interference (RNAi) causes a reduction of cap cells. (A) Fusion of germarium and follicles is typical of a *N* loss-of-function phenotype. (B) This germarium has only two cap cells (one visible) and one GSC. Note that the cap cell (open arrowhead) expresses Tj, is round and not aligned with the TF. (C) Absence of cap cells is accompanied by a lack of GSCs. A differentiating germline cyst (stippled outline) is located at the tip of the germarium adjacent to ectopically located escort cells (EsC). (D-F) N depletion in combination with Tj overexpression causes a phenotype similar to *N* knockdown alone: compare panel D with A, E with B, and F with C. (G-I‴) Ovaries with *N tj* double-knockdown show a combination of *N* and *tj* loss-of-function characteristics: all anterior niche cells display the TF fate but their number is reduced. (G) The ovary contains only TFs, surrounded by ovariole sheaths, and a small germline cluster. (H-I‴) All cells in the elongated TFs display the shape and markers of TF cells. (J) The graph compares the number of different cells in the GSC niche of ovaries that have reduced N expression combined with endogenous, reduced or increased levels of Tj expression. *UAS*-constructs were driven by *tj-Gal4*, and *w* flies served as a control. Mean + s.d. are indicated. *n*, sample size. (**K-T**) The effects of altered Tj expression in ovaries that express constitutively active N (N^intra^) in a *tj*-specific pattern. In contrast to a *tj* overexpressing ovariole that has a normal-sized niche (K), germaria that expressed N^intra^ either alone (L) or in combination with transgenic Tj (M) display a massively increased pool of cap cells, as highlighted by strong expression of Bab2, but lack Vasa-positive germ cells. (N) A Tj-depleted ovary with abnormally long TFs (yellow stippled lines). Cap cells are absent. Sheath cells fill the space between the elongated TFs. (O) A N^intra^ expressing ovariole with a normally looking TF and an increased number of cap cells that contact a cluster of GSC-like cells (pink stippled line). (P) A rudimentary ovary, caused by depletion of Tj and expression of N^intra^ that only consists of a group of abnormally elongated TFs attached to fatbody. Cap cells and sheath cells are absent. (Q) Abnormally elongated TFs in an ovary that co-expresses *tj*^*RNAi*^ and GFP. The GFP-positive TF cells represent transformed cap cells. (R) In N^intra^ GFP co-expressing ovarioles, GFP is seen in cap cells but not in TFs. LamC staining in cap cells is visibly weaker than in TF cells. (S) A very long TF surrounded by fatbody cells in an ovary that co-expresses *tj*^*RNAi*^ and N^intra^. Strong staining of LamC is seen throughout the TF. (T) The graph compares the number of cells per TF between the indicated genotypes. Mean + s.d. are indicated. *n*, sample size. (U) Schematic drawing illustrates the niche defects resulting from altered Tj and N expression. Anterior is up in all images. Scale bars: 25 μm in A,D,G, and 10 μm in all other images.

Mutants with reduced N activity displayed a decrease in the number of cap cells [8,31,32]. Similarly, knocking down N by *tj*-Gal4-driven expression of *UAS*-*N*^*RNAi*^, which caused a typical *N* loss-of-function phenotype with fused follicles (Fig 8A) [56], reduced the average number of cap cells per germarium from a normal complement of 6 down to 2 (Fig 8B and 8J). Accordingly, the average number of GSCs dropped from 3 in control germaria to 0.8 in *N*^*RNAi*^ germaria (Fig 8J). When all cap cells were missing, GSCs were absent, and escort cells were misplaced to the tip of the germarium, where they made contact with the TF and a differentiating germline cyst (Fig 8C). Although the number of cap cells was reduced, the number of TF cells was normal in N depleted ovarioles (Fig 8J), indicating that the loss of cap cells is not due to a cap cell to TF cell fate change. This shows that the *tj* and *N* loss-of function phenotypes are different.

As both Tj and N are required for cap cell formation, we asked if and how their functions might be related. First, we investigated whether their expression is dependent on each other. To determine whether the expression of Tj depends on N signaling we checked Tj expression in *N*^*RNAi*^ ovaries. However, we could not separate a direct effect on Tj from an effect on cap cells. Remaining cap cells in *N*^*RNAi*^ ovaries always expressed Tj, and at an apparently normal level (Fig 8B). That existing cap cells remained in the germarium and were not recruited into TFs also suggests that the expression level of Tj is not affected. Although this argues against Tj being a downstream target of the N pathway, it cannot be excluded that remaining N activity in *N*^*RNAi*^ ovaries can enable the formation of a few cap cells with full expression of Tj.

To test whether Tj influences the expression of N signaling components, we evaluated the expression pattern of N, its ligand Dl, and its target and effector Enhancer of split (E(spl)) in *tj*^null^ ovaries at the prepupal stage when anterior niches have formed (S4 Fig). Dl staining was much stronger in TFs than in cap cells of control ovaries (S4A Fig) [8]. Dl staining in the upper half of TFs in *tj*^null^ ovaries was as robust as in controls. Interestingly, however, Dl staining in the lower portion of the stalk, which is composed of transformed cap cells in *tj*^null^ ovaries, was as weak as in cap cells of controls (S4B Fig). Thus, in contrast to all other tested markers, Dl expression appears not to have changed in the transformed cap cells, indicating that Dl expression in niche cells is not regulated by Tj. Expression of N protein and an *E(spl)* expression reporter (*E(spl)mß*-CD2), which can be used to detect activity of the N pathway in niche cells [8], appeared to be rather homogeneous throughout the anterior niche cells of *tj* mutant ovaries similar to wild-type ovaries (S4C and S4D Fig). Thus, Tj appears not to affect the activity of the N pathway in cap cells. Taken together, our expression analysis is consistent with Tj acting downstream or in parallel to the N pathway in the formation of cap cells.

To further analyze the relationship between Tj and N, we looked for genetic interactions by changing their expression level either in the same or opposite direction. Overexpressing Tj in its endogenous pattern, including cap and escort cells, by driving expression of *UAS*-*tj* with *tj*-Gal4 did not cause any obvious defects in the stem cell niche (Fig 8K), although it led to defects at later stages of oogenesis. The *N*^*RNAi*^ phenotype prevailed in the presence of increased Tj expression (Fig 8D–8F and 8J), suggesting that Tj cannot rescue cap cells in the absence of N.

To achieve a double knockdown of Tj and N, we used a *UAS*-*tj*^*RNAi*^ transgene that strongly reduces Tj expression [57]. When driven with *tj*-Gal4, the *tj*^*RNAi*^ knockdown was variable but consistently strong and frequently caused a phenotype that was indistinguishable from a *tj*^null^ ovary phenotype (Fig 8N). *tj N* double-knockdown ovaries largely resembled *tj*^null^ ovaries, with TFs and ovariole sheaths remaining and all other cell types drastically reduced or missing (Fig 8G). Not surprisingly, cap cells were absent (Fig 8J). Interestingly, however, *tj*^*RNAi*^ *N*^*RNAi*^ ovaries did not have the extended TFs that are the hallmark of *tj* mutant ovaries (Fig 8G). With an average of 9.1 cells, TFs of *tj*^*RNAi*^ *N*^*RNAi*^ ovaries were considerably shorter than those of *tj*^*RNAi*^ ovaries, which had an average of 16 cells similar to *tj* mutant ovaries (compare Fig 8G with 8N and 8Q). Although the number of TF cells per stack was highly variable in *tj*^*RNAi*^ *N*^*RNAi*^ ovaries (Fig 8J), 42% of the TFs had more than 8 cells per stack and were therefore longer than those of control ovaries (average of 7.8 cells), resembling more the combined number of TF and cap cells in the niches of *N*^*RNAi*^ ovaries (average of 9.9 cells) (Fig 8J). All cells in these elongated stalks of *tj*^*RNAi*^ *N*^*RNAi*^ ovaries had a TF cell identity based on morphology and marker expression (Fig 8H and 8I‴). We propose that cap cells that remained after strong reduction of N expression acquired the TF cell fate due to the loss of Tj. Further analysis of ovaries with *tj N* double-knockdown revealed that the total number of TF stalks was strongly reduced, with an average of 6.2 (n = 12), compared to 16.1 in *N*^*RNAi*^ ovaries (n = 14) and 19.3 in *tj*^*RNAi*^ ovaries (n = 9), and that most ovaries contained several unusually short TFs with less than 7 TF cells. This suggests, that loss of Tj and/or N does not only affect cap cell formation but that their combined loss affects TF cell formation as well.

Next, we investigated the effects of increased or decreased Tj expression on the phenotype caused by N^intra^, which constitutively activates N signaling. Overexpression of Tj did not appear to affect the number of cap cells (Fig 8K), whereas driving *UAS*-*N*^*intra*^ with *tj*-Gal4 led to a large increase of cap cells (Fig 8L), similar to what had been reported previously for N^intra^ expression with another somatic driver [8]. Co-expression of N^intra^ and transgenic Tj resulted in the same phenotype (Fig 8M). For both genotypes, we observed two different types of germaria. Some germaria contained a cluster of undifferentiated germ cells that resembled GSCs (Fig 8O), consistent with a previous report [8], whereas other germaria were devoid of germ cells despite a large aggregate of cap cells (Fig 8L and 8M).

We were particularly interested in the phenotype of *N*^*intra*^ *tj*^*RNAi*^ ovaries. If N determines the number of cap cells while Tj controls their identity, one might expect that all *N*^*intra*^-induced additional cap cells become TF cells in the absence of Tj, causing even longer TFs than when Tj alone is lost. The phenotype of *N*^*intra*^ *tj*^*RNAi*^ ovaries was variable, consistent with the variability in the individual *tj*^*RNAi*^ and *N*^*intra*^ phenotypes but the defects were considerably more severe (Fig 8N–8P). Most *N*^*intra*^ *tj*^*RNAi*^ ovaries were extremely small, not connected to an oviduct and often attached to the gut, and seemed to consist largely of TFs and a few germline cells embedded in fatbody (Fig 8P). In contrast to *tj*^*RNAi*^ or *N*^*intra*^ ovaries, the epithelial sheaths were missing and TFs were located side-by-side (Fig 8N–8P). In the most severe cases, *N*^*intra*^ *tj*^*RNAi*^ ovaries consisted only of TFs (Fig 8P). To account for the two UAS-constructs in *N*^*intra*^ *tj*^*RNAi*^ ovaries, we co-expressed a second UAS-construct together with *tj*^*RNAi*^ or *N*^*intra*^ in our controls, which expressed GFP (Fig 8Q and 8R). This did not appear to influence the mutant phenotype but showed that *tj*-Gal4 is active in the lower half of the extended TFs in *tj*^*RNAi*^ ovaries (Fig 8Q), and is excluded from the TF but present in cap cells of *N*^*intra*^ ovarioles (Fig 8R), as expected. Notably, TFs in *N*^*intra*^ *tj*^*RNAi*^ ovaries were always considerably longer than in *N*^*intra*^ ovaries (Fig 8O, 8P and 8R–8T), although they were on average slightly shorter than in *tj*^*RNAi*^ ovaries (Fig 8N, 8P and 8T). Fig 8S shows a rare example of a particularly long TF in a *N*^*intra*^ *tj*^*RNAi*^ ovary. Taken together, these findings suggest that the *tj*^*RNAi*^ niche phenotype is epistatic to the *N*^*intra*^ niche phenotype. The effects on cap cells in response to alterations in Tj and N expression are summarized in Fig 8U.

## Discussion

### Tj controls the capacity of the somatic niche for GSCs

Loss of Tj has a profound negative effect on the establishment, number, and maintenance of GSCs. Effects of Tj on the germline were previously shown to be indirect as Tj is neither expressed nor cell-autonomously required in the germline [36]. Therefore, we propose that the dramatic change in the structure of the somatic niche affects GSCs when Tj function is compromised. An inverse causal relationship, where a reduced number of GSCs would trigger the somatic niche defects was ruled out by showing that cap cells can still look and behave normally in the absence of any germ cells. We conclude that Tj controls GSCs indirectly by controlling somatic cell fate and cell arrangement in the stem cell niche.

By controlling the morphology and behavior of the cap cells, Tj regulates the GSC-carrying capacity of the niche. When Tj expression was moderately reduced, the number of GSCs per niche was reduced, with the remaining GSC properly maintained over several weeks. The decrease of GSCs per niche correlated with a decrease of cap cells in the germarium. Two cap cells were on average required to sustain one GSC, similar to what has been proposed for a wild-type ovary [6,8]. Our data indicate that the reduced niche capacity is due to a reduction in the available contact surface between cap cells and GSCs. Tj-depleted cap cells that convert from forming a cluster inside the germarium to forming a stalk outside the germarium minimize their availability for GSC attachment. A connection between the GSC-cap cell contact area and niche capacity is similarly reflected in the increased number of GSCs that accompanies an increase in cap cell size due to loss of RanBPM [25]. Here, we show that the spatial arrangement of the cap cells has a crucial impact on the number of stem cells per niche.

When Tj function was completely abolished, the number of GSCs was drastically reduced, as expected in the absence of cap cells. The very few pMad-positive GSC-like cells in *tj* mutant prepupal ovaries were always associated with a TF, suggesting that TFs might temporarily provide enough Dpp to activate Mad in a few germline cells, consistent with the finding that Dpp is expressed in TFs at the late larval stage [17,58]. This is not sufficient, however, to maintain GSCs and adult ovaries rarely contain pMad-positive germline cells. This is in agreement with the finding that Dpp is not detected in adult TFs [6], and corroborates that cap cells are required for GSC maintenance. In addition, the rapid loss of the entire germ cell pool in Tj-depleted ovaries during the pupal stage might be precipitated by loss or defects in escort cells. Escort cell precursors are not properly intermingled with germ cells at the larval stage and differentiated escort cells appear to be missing in adult ovaries that lack Tj [36]. As escort cells are crucial for germ cell differentiation [59–62], the defect in escort cell differentiation could be responsible for the demise of the germline in *tj* mutants.

We discovered that GSCs have broad cellular protrusions, which they use to reach and tightly ensheath the accessible surface of cap cells. In wild type, relatively short protrusions are sufficient to make extensive contact with more than one cap cell. However, when cap cells formed a stalk, GSCs were often observed to produce unusually long extensions that allowed them not only to contact the immediate cap cell neighbor but also a more distantly located cap cell. This suggests that GSCs respond to a chemotactic signal from cap cells and send protrusions toward this signal. It remains to be investigated whether this is a response to Dpp signaling or signaling through another pathway. The importance of cellular protrusions in signaling events in the stem cell niche has recently come to light with the discovery of nanotubes that mediate Dpp signaling between GSCs and hub cells in the *Drosophila* testes [63], and cytonemes that contribute to Hh signaling from cap to escort cells in the ovary [12].

### Tj is essential for the specification of cap cells

Our analysis shows that Tj is required for the specification of cap cells. In the absence of Tj function, additional TF cells form at the expense of cap cells, resulting in unusually long TFs while the cap cell fate is not established. Whereas the formation of cap cell precursors appears not to require Tj, this transcription factor is essential for the ability of these precursors to take on the cap cell fate and to prevent the TF cell fate that is otherwise adopted as a default state. The following findings support this conclusion: (i) In the absence of Tj function, cap cells were missing while additional cells that displayed TF cell-characteristic morphology, behavior and marker expression were integrated into the TF. The number of additional TF cells was comparable to the normal number of cap cells. (ii) Prospective cap cells cell-autonomously adopted a TF-specific morphology and behavior in the absence of functional Tj. (iii) A hypomorphic *tj* mutant provided direct evidence for the incorporation of cap cells into TFs, forming the basal portion of these stalks. (iv) Ectopic expression of Tj in TF cells caused a change toward cap cell-typical marker expression and morphology. Together, these data demonstrate that Tj promotes cap cell specification.

The expression pattern of Tj supports the notion that Tj has a function in cap cells but not in TF cells. Tj is continuously expressed in cap cells [36; this study]. Tj is also present in the anterior interstitial cells of the larval ovary [36,44], which are thought to develop into cap cells [14]. In contrast, Tj is neither detected in the cell population that gives rise to TFs during 3rd larval instar, nor in differentiated TFs [28,36]. Interestingly, we found that even in the absence of Tj function, the *tj* gene remains differentially expressed in the anterior niche, being inactive in regular TF cells but active in the additional TF cells, which form the apical and basal portion of a TF, respectively. This differential expression of Tj indicates that a regionally or temporally regulated mechanism operates upstream of Tj that initiates differences in anterior niche cells. Although it is conspicuous that Tj expression from 3rd instar onwards is restricted to cells that are in direct contact with germline cells, which includes cap cells but excludes TF cells, it has previously been shown that Tj expression is not dependent on the germline [36,43]. This suggests that a soma-specific mechanism is responsible for the differential expression of Tj in anterior niche cells. Interestingly, a recent study uncovered the importance of Hh signaling from TFs to neighboring interstitial cells in the larval ovary and proposes that *tj* is a direct target of the Hh signaling pathway [30].

### Differential expression of Tj defines different cell fates in the GSC niche

Our findings suggest the presence of a new cell type in the GSC niche that we named 'transition cell' as it is located between the cap cell cluster and the TF, connecting these two structures of the niche. Notably, the one or occasionally two transition cells have the morphology of TF cells and align with neighboring TF cells despite displaying a cap cell-like marker profile that includes the expression of Tj—although Tj expression is substantially lower than in cap cells. Interestingly, cap cells from ovaries with reduced Tj expression (*tj*^hypo^) similarly displayed a TF cell-like morphology and behavior while their expression profile remained cap cell-like. A similar, although weaker effect was noted in a *tj* hemizygous condition, suggesting that Tj function is haplo-insufficient in cap cells. Thus, when Tj levels are reduced, cap cells adopt very similar molecular and morphogenetic properties as the transition cell in a wild-type niche, and might have adopted this cell fate.

**Fig 9:**
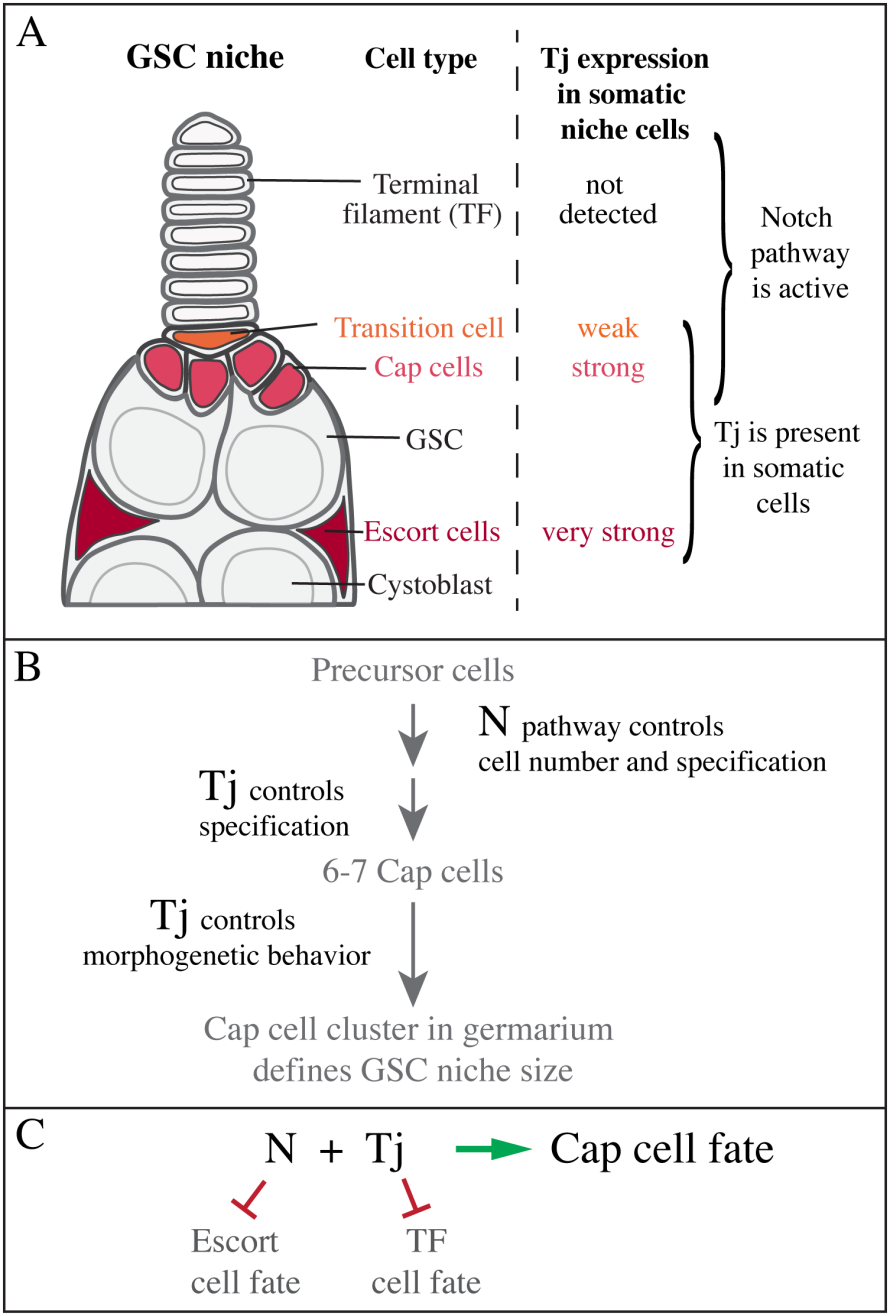
Model of Tj function in the GSC niche. (**A**) Tj is expressed at different levels in the somatic cell types of the GSC niche. Cap cells contain both Tj and activated N in contrast to TF and escort cells. (**B,C**) The combined action of Tj and N is required to establish the cap cell fate.

Together, our findings indicate that Tj has an important role in the establishment of three cell types in the GSC niche: TF cells, transition cells, and cap cells. As lack of Tj function seems to cause a transformation of cap and transition cells into TF cells, and a mild reduction of Tj a cap to transition cell transformation, we propose that different Tj expression levels establish different cell fates and morphogenetic traits. We propose that a high concentration of Tj leads to the formation of cap cells and a lower concentration to the formation of the transition cell, whereas absence of Tj is required for the formation of TF cells (Fig 9A). This model implies that different levels of Tj have different effects on target genes. We predict that Tj has at least one target gene that only responds to high levels of Tj and that specifically controls the morphogenetic behavior of cap cells, allowing them to adopt a round morphology and organize into a cell cluster. Whether this relates to an effect of Tj on the expression of adhesion molecules as observed in other gonadal tissues [36,42,57,64, 30] awaits further analysis.

### Combined action of Tj and the N pathway is required for cap cell formation

Our study identifies Tj as essential for cap cell formation. In addition, this process depends on the N pathway [8,31,32]. Therefore, we wondered how the functions of Tj and N in cap cell formation relate to each other (Fig 9). A comparison between the loss and gain-of-function phenotypes suggests that Tj and N have different functions in the establishment of cap cells. In the absence of Tj function, cap cell precursor cells are present but take on the fate of TF cells, whereas depletion of N leads to a loss of cap cells but does not cause the formation of additional TF cells. Ectopic activation of N can induce a strong increase in the number of cap cells, whereas overexpression of Tj did not appear to affect the number of cap cells. Therefore, both factors are important for cap cell formation but contribute differently to this process. The questions then are: What is the respective contribution of Tj and N to cap cell formation, and how are their functions related?

The function of N in cap cell formation is still not fully understood. Our observation that depletion of N reduces the number of cap cells confirms previous findings [8,32,65]. However, neither in our nor any previously published experiments were cap cells lost completely when the N pathway was compromised, and it remains therefore unclear whether N is de facto essential for cap cell formation or primarily functions in regulating the size of the cap cell pool. Interestingly, evidence amounts to a function of the N pathway in a decision between the cap cell and escort cell fate: First, Dl signal from TF cells activates the N pathway in adjacent interstitial cells, inducing them as cap cells, whereas the remaining interstitial cells are thought to develop into escort cells [8,32]. Second, escort cells expressing activated N can develop into cap cells [31,65]. Third, when we used *tj*-Gal4 to express active N in interstitial cells, the number of cap cells dramatically increased while the escort cell region became smaller, and some germaria seemed to lack escort cells all together. These germaria also lacked germline cells, although a larger pool of cap cells was expected to increase the number of GSCs [8,31]. However, the absence of germline cells is consistent with an absence of escort cells, as escort cells have been shown to be important for maintaining the germline [60]. Together, these observations support the hypothesis that N is involved in a cap cell versus escort cell fate decision, and suggest that the N pathway might promote the formation of cap cells by inhibiting the escort cell fate.

To determine how the functions of Tj and N depend on each other we looked for genetic interactions. The N pathway seems to be still functional in *tj* mutants. First, the expression of N and Dl appeared unaffected and *E(spl)* was activated in the additional TF cells (= transformed cap cells) similarly to normal cap cells. Second, the formation of additional TF cells in the absence of Tj depended on the presence of N, as only very few additional TF cells formed in a N compromised background. These findings indicate that the N pathway is still active in cap cell precursors when Tj function is abolished. This together with the observation that constitutively active N cannot suppress the *tj* mutant phenotype suggests that Tj does not act upstream of N in regulating cap cell fate.

Therefore, we asked whether Tj might operate downstream of N. We did not detect a loss of Tj upon N depletion, and this together with the finding that Tj is expressed in all interstitial cells, and not only in those that receive Dl signaling argues against a requirement of N signaling for *tj* expression. If at all, one would expect *tj* to be negatively regulated by N as cap cells express a lower level of Tj than escort cells. The maintenance of somatic cell types in *N* mutant ovaries that are lost in *tj* mutant ovaries, including the escort cells is also not consistent with a linear relationship. Nevertheless, the ability of Tj to promote the formation of cap cells appears to depend on the activity of the N pathway in cap cell precursors. Again, this is suggested by the finding that when N and Tj were both compromised, the number of additional TF cells were much smaller than when N was fully active. Therefore, we propose that N activity sets aside a pool of percursor cells that in the presence of Tj take on the cap cell fate, and in its absence the TF fate (Fig 9B).

Similar to the ovary, N is important for the formation of the GSC niche (= hub) in the *Drosophila* testis [66,67]. Interestingly, N contributes to hub cell specification by downregulating the expression level of Tj [42]. Not only is the hub still present in *tj* mutant testes [36] but additionally, ectopic hub cells form in the absence of Tj [42]. Thus, Tj seems to have opposing functions in testes and ovaries, suppressing the niche cell fate in the testis [42], while promoting it in the ovary.

The interplay between Tj and N seems not restricted to the cap cell fate in the ovary. Whereas neither factor alone is required for TF cell formation, as TF cells formed normally in the absence of either Tj or N, the combined loss of Tj and N led to a strong reduction in the number of TFs and number of TF cells within stalks. This suggests that their combined action is already required at an earlier stage of ovary development, when Tj is still expressed in all somatic cells of the ovary [44]. Moreover, Tj knockdown combined with expression of activated N caused TF cells to be the only cell type remaining of the ovary, indicating that several cell types in the ovary require proper input from both factors. Taken together, our findings support a model, in which both Tj and N operate together to promote the cap cell fate but have separate functions. We propose that Tj and N promote the cap cell fate by blocking the TF cell fate and escort cell fate, respectively, and that the combined actions of Tj and the N pathway are required to establish the cap cell fate (Fig 9C).

## Materials and methods

### Fly stocks

*tj*^*eo2*^ (amorphic) [36,40], *tj*^*z4735*^ (amorphic) [36,41], *tj*^*39*^ (hypomorphic, see below), *tj-Gal4* (hypomorphic, see below), *tj*^*Df1*^ (molecular null, see below), and *UAS*-*tj*^*RNAi*^ [57] (NIG-Fly Stock Center] were used for *tj* loss-of-function analysis. *UAS-tj*^*1(2)*^ and *UAS-tj*^*6(3)*^ (*UAS-tj*; full-length *tj* coding sequence and 3'UTR) [57] were used for ectopic and overexpression of Tj. *UAS-N*^*754*.*BF*^ (*UAS-N*^*intra*^ on 3rd) [68] and *P{TRIP*.*JF02959}attP2* (*UAS-N*^*RNAi*^) [69] (Bloomington *Drosophila* Stock Center (BDSC)] were used for *N* loss- and gain-of-function experiments. *tud*^*1*^ and *tud*^*b45*^ [70] (gift from M. Van Doren), and *osk*^*301*^ [54] were used to generate flies without a germline. We used *tj*-*Gal4* (*P{GawB}NP1624*) [71,72] (Kyoto Stock Center) to drive expression in cap and escort cells and their larval progenitors, and the FLPout cassette [73] (BDSC) for clonal expression in TF cells. *tj*^*z4735*^ mutant cell clones were induced by mitotic recombination using *hs-FLP1* and *FRT40A* (BDSC). *UAS-GFP*.*S65T* and *Ubi-GFP* (BDSC) were used as clonal cell markers. *nos-Gal4* (BDSC) was used to drive *UAS-GAP43-mEos* (BDSC) in germline cells. The enhancer reporter lines *bab*^*A128*^ (*bab-lacZ*) [15,74], *P{PZ}1444* (*1444-lacZ*) [55], *P{A92}LB27* (*LB27-lacZ*) [16], *P{lacW}B1-93F (B1-lacZ*) [15,56], and *bamP*^*702*^*-GFP* (*bam-GFP*) [75] were used as cell type specific markers. *E(spl)mß-CD2* was used to monitor activity of the N pathway [8,76] (gift from D. Drummond-Barbosa). We generated the following recombinant chromosomes: *tj*^*eo2*^ *1444-lacZ*, *UAS-tj*^*1(2)*^ *1444-lacZ*, and *UAS-tj*^*1(2)*^ *UAS-GFP* for functional analysis of *tj*, and *UAS-tj*^*6(3)*^ *UAS-N*^*RNAi*^, *UAS-tj*^*6(3)*^ *UAS-N*^*intra*^, *UAS*-*tj*^*RNAi*^ *UAS-N*^*RNAi*^, *UAS*-*tj*^*RNAi*^ *UAS-N*^*intra*^, and *tj-Gal4 UAS-GFPnls* to test genetic interactions between *tj* and *N*. Oregon R, *w*, or *y w* were used as a genetic background. The copy number of all genetic markers, such as enhancer reporters was identical between control and experimental animals.

### Generation of new *tj* mutations

*tj*^*Df1*^, a transcriptional null mutation is a genomic deletion of 13.3 kb (2L:19464294–19477599), beginning 286 bp upstream of the *tj* start codon and ending 9.8 kb downstream of the *tj* transcription unit (S1A Fig). This mutation deletes the complete coding and 3' UTR sequences of *tj*, and three predicted RNA coding genes. *tj*^*Df1*^ was generated by FLP-mediated recombination between the FRT elements of the transposable elements *P{XP}d06467* and *PBac{WH}f02713* [77,78] (Exelixis Collection at the Harvard Medical School), using the technique described by Parks et al. [79]. We screened for recombinant flies by eye color as recombinant chromosomes containing a deletion were expected to carry two mini-*white* genes. Recombination was confirmed by PCR analysis, using genomic DNA from homozygous *tj*^*Df1*^ flies. Primer pair AGCGAATGGTGGCGTTCGAGCTC—ACCACCTTATGTTATTTCATCAT confirmed the presence of the 3' end of *P{XP}d06467*, primer pair CCTCGATATACAGACCGATAA—AGCCAAATGAACTGCCCGCT the presence of the 3' end of *PBac{WH}f02713*, and primer pair GACCTTTGAAACCACCCACTAAC—GTGGTGTGCGTAAGTCTGAGC the absence of *tj*-specific sequences. *tj*^*Df1*^ is homozygous viable, but both female and male sterile.

*tj*^*39*^, a weak hypomorphic allele was generated in a P element excision mutagenesis, using *tj-Gal4* (*P{GawB}NP1624*), which is located in the 5' UTR of *tj*, 0.7 kb upstream of the translation start site [72], as a starter line. *tj*^*39*^ caused strongly reduced fertility in trans to *tj*^*eo2*^ (approximately 20% of the fertility of the *tj*^*eo2*^/+ control), whereas 29 other excision mutations were fully fertile in trans to *tj*^*eo2*^. PCR analysis, using genomic DNA of homozygous mutant *tj*^*39*^ flies and four primer (P) pairs (P2: GCTCTTGCACAGTGGTCGAG—P1: ACCACCTTATGTTATTTCATCAT, P1: ACCACCTTATGTTATTTCATCAT—P3: GTGTCGTTTATGGTGGGATC, and P2: GCTCTTGCACAGTGGTCGAG—P4: GAACTCCTGTTGGAAACGTG showed that the genomic sequences flanking the insertion site are still present and revealed a partially excised P element (the 3' end is still present). Sequencing the PCR-amplified *tj* coding region, using primers described in Li et al. [36], confirmed that the *tj* open reading frame is intact, suggesting that the remaining P element impairs *tj* expression at the transcriptional or translational level. Subsequent tests revealed that *tj-Gal4* itself is a weak hypomorphic allele of *tj*, causing a similar phenotype in trans to a *tj* null allele as its derivative *tj*^*39*^. *tj*^*39*^ tested positively for Gal4 activity.

### Experimental conditions

Flies were raised and maintained at 25°C on standard *Drosophila* medium supplemented with yeast pellets. Ovaries were extracted from 1–4 hour old prepupae, two-day-old pupae, or 1–2 day old yeast-fed adult females, which had been kept in the company of males unless indicated otherwise. Staging, dissection, and processing of prepupal and pupal ovaries were done as described in Godt and Laski [15]. For the aging experiment, female flies were collected and separated from males within 24 hours of eclosure, and were transferred every day to a new food vial (supplemented with yeast pellets) until they were dissected 2, 7, 14, and 22 days after eclosure. All experiments were independently repeated at least twice.

Clonal analysis: (1) *tj* mutant cap cell clones were induced in *y w hsFlp1/+; tj*^*z4735*^ *FRT40A/ P{Ubi-GFP*.*D}33 P{Ubi-GFP*.*D}38 FRT40A* larvae by three 2-hour heat shocks at 37°C during early to mid 3rd instar (at 72–74, 82–84, and 90–92 hours after egg deposition). Animals were reared at 25°C to adulthood and ovaries dissected from 2-day old females. (2) To generate Tj-expressing cell clones in TFs we used the following genotypes: *y w hsFlp1/+; UAS-tj*^*1(2)*^ */+; Act5C>CD2>Gal4/+*, or *y w hsFlp1/+; UAS-tj*^*1(2)*^ *1444-lacZ /+; Act5C>CD2>Gal4/+*, or *y w hsFlp1/+; UAS-tj*^*1(2)*^*/+; Act5C>CD2>Gal4/LB27-lacZ*, or *y w hsFlp1/+; UAS-tj*^*1(2)*^ *UAS-GFP/+; Act5C>CD2>Gal4/+*. Flies of the genotype *y w hsFlp1/+; UAS-GFP/+; Act5C>CD2>Gal4/+* were used as a control. Early 3rd instar larvae (72 ^+^/-1.5 hours at 25°C after egg deposition) were heat shocked at 37°C for 11 minutes, cooled down to 25°C for 10 minutes in a water bath, and reared at 25°C to adulthood. Ectopic expression of Tj caused a relatively high degree of lethality in larvae and pupae, and ovaries were extracted from escaper flies. To measure the height and width of a TF cell, a line through the center of the cell was drawn along the anterior-posterior axis and perpendicular to it, respectively, and ßPS integrin was used to recognize the plasma membrane.

### Tissue immunostaining

The following primary antibodies were used: guinea-pig anti-Tj (G5 or GP6, 1:5000) [57], rat anti-Bab2 (R10, 1:3000; or R7, 1:2000) [20], rabbit anti-Vasa (1:2000) [80], rabbit anti-Vasa (d-260, 1:500; Santa Cruz Biotechnology), chicken anti-Vasa (1:5000; gift from K. Howard and M. Van Doren), rabbit anti-α-spectrin (#254, 1:1000; gift from D. Branton), mouse anti-LamC (LC28.26, 1:50), mouse anti-Hts (1B1, 1:5), mouse anti-N (C17.9C6, 1:5; C458.2H, 1:5), mouse anti-Dl (C594.9B, 1:5), mouse anti-Engrailed (4D9, 1:5), and mouse anti-ßPS integrin (CF.6G11, 1:10) (Developmental Studies Hybridoma Bank), rabbit anti-pMad (PS1, 1:250; gift from T. Tabata) [81], rabbit anti-pMad (pSmad1/5, 41D10, 1:100; Cell Signalling), rabbit anti-ß-galactosidase (1:1500; MP Biomedicals), and rabbit anti-GFP (1:100; BD Biosciences). Secondary antibodies (1:400) were conjugated either to Cy3, Cy5 (Jackson Immuno Research Laboratories), Alexa-405, Alexa-555, Alexa-488, or Alexa-647 (Molecular Probes, Life Technologies). Ovaries were mounted in Vectashield (Vector Laboratories).

### Imaging

All imaging was done with a 40x/1.4 Plan-Apo objective, using confocal laser scanning microscopes LSM510 (Carl Zeiss Microscopy) and Leica TCS SP8 (Leica Microsystems) at RT. A zoom factor of 4–5 was used to image individual stem cell niches. Images represent either individual confocal sections or projections of 2–3 sections that were chosen from Z-stacks (1 μm intervals), which were routinely acquired of all studied germaria. Image analysis, cell counts and cell shape measurements were done by evaluating Z-stacks, using the LSM 5 Image Browser and (Carl Zeiss Microscopy) and Leica LAS X software (Leica Microsystems). Images were processed with Adobe Photoshop and Illustrator CS5 and CS6 (Adobe Software).

### Statistical analysis

Unpaired, two-tailed Student’s t-tests or one-way ANOVA tests were used for statistical analysis. Prism 6 (GraphPad Software) was used for statistical tests, and Prism 6 and Illustrator CS6 for the generation of graphs.

## Supporting information

S1 Fig Molecular characterization of new *tj* mutations. (**A**) Map of the genomic region encompassing the *tj* locus, showing the insertions used to generate the *tj*^*Df1*^ deletion in blue, and the insertion used to generate *tj*^*39*^ in yellow. *tj*^*Df1*^ deletes most of the transcription unit of *tj*, including the whole *tj* coding sequence and 3'UTR, and three predicted RNA coding genes. *tj*^*39*^ contains a partial P element in the promoter region of *tj*. (**B**) Analysis of the *tj*^*39*^ mutation by PCR shows that the 5' region of the *tj-Gal4* insertion was excised but the flanking genomic regions remained unaffected. Target regions for primers P1-4 are indicated by arrows and listed in Material and Methods. (**C**) Immunoblot analysis of Tj proteins encoded by wild-type and *tj* mutant alleles in adult ovaries. The blot was probed with anti-Tj (red) and anti-Armadillo (Arm, green) antibodies. Wild-type Tj protein (Tj^full-length^) runs at ~72 kDa, which is higher than expected based on its sequence (expected molecular weight: 54.3 kDa). *tj*^*eo2*^, which has a premature stop codon [36], produces a truncated non-functional protein (Tj^C384stop^). The presence of the *tj*^*39*^ allele in *tj*^*39*^*/tj*^*eo2*^ ovaries caused a reduction in the amount of the Tj^full-length^ protein but not of the Tj^C384stop^ protein in comparison to *tj*^*eo2*^*/+* ovaries. (**C'**) Quantification of Tj^full-length^ protein, based on three immunoblots, including the one shown in (C), showing mean + s.d. The Tj signal was normalized to the Arm signal that was used as a loading control, and the Tj signal intensity from the wild-type (+/+) lane was set to 100%.
(TIF)

S2 Fig In *tj* mutants, additional TF cells form at the expense of cap cells. Images show the adult GSC niche. An arrowhead marks the TF/germarium boundary (A-I), a bracket the cap cell cluster (A,C,E), and an arrow the anterior-most Tj-positive cell in a TF (B,F,G). (**A-D**) *B1-lacZ* and LamC are strongly expressed in TF cells and weakly in Tj-positive cap cells in the controls (A,C) and in the *tj*^*39*^*/tj*^*eo2*^ ovary (*tj*^hypo^) (B). In contrast, both markers are strongly expressed throughout the extended TF (stippled line) of a *tj*^*eo2*^*/tj*^*eo2*^ mutant (*tj*^null^) (D). (**E-G**) *LB27-lacZ*, which is exclusively detected in TF cells and absent from Tj-positive cap cells in the control (E), is sometimes seen in Tj-positive cap cells outside the germarium (asterisks) in a *tj*^*39*^*/tj*^*eo2*^ mutant (*tj*^hypo^) (F), and always found in the Tj-positive cells of the extended TF in a *tj*^*z4735*^*/tj*^*eo2*^ ovary (*tj*^null^) (G). *tj*^*z4735*^ produces a detectable Tj mutant isoform. (**H,I**) *tj*^*null*^ mutant cell clones (homozygous for *tj*^*z4735*^) in the anterior niche. Images show projections of full Z-stacks of the GSC niches depicted in Fig 2H and 2I. Mosaic TFs contain *tj* mutant cells that lack GFP in the posterior portion. As these cells express Tj (mutant isoform), they represent transformed cap cells that are ectopically located in the TF (asterisks). Note that all escort cells in the vicinity of the cap cells express GFP. Genotypic markers: *B1-lacZ/+* (A,B) or *B1-lacZ* (C,D), *LB27-lacZ/+* (E-G), *Ubi-GFP* (H,I). Anterior is up in all panels. Scale bars: 10 μm.
(TIF)

S3 Fig GSCs are maintained in *tj* hypomorphic mutant ovaries. Vasa (blue) marks germline cells and LamC (green) labels TFs in all panels. Tj (green in A-F) marks cap, escort, and follicle cells, *bam*-GFP (red/white in A-F) marks early differentiating germ cells, and pMad (red/white in G,H) labels GSCs. The germarium-TF boundary is marked by an arrowhead (E-H). GSCs are marked by an asterisk. (**A-D**) Similar to the control (wt), *tj*^*eo2*^*/tj*^*39*^ ovarioles (*tj*^hypo^) contain a germarium (Gm) followed by a series of growing follicles in 2-day-old (A,B) and 3-week-old females (C,D), indicating the presence of at least one GSC. (**E-H**) The maintenance of two GSCs in a wild-type ovariole and one GSC in a *tj* mutant ovariole is indicated by the absence of *bam*-GFP (E,F) and the presence of pMad (G,H) in 3 and 2 week-old females, respectively. Genotypic marker: *bam-GFP* (A-F). Scale bars: 50 μm in A-D; 10 μm in E-H.
(TIF)

S4 Fig Expression of N pathway components is comparable in *tj* mutant and wild-type GSC niches. Images show the GSC niche in prepupal ovaries. Tj marks cap cells (brackets) and somatic cells that are intermingled with germ cells, *bab-lacZ* labels TF and cap cells, and Vasa labels germ cells. (**A,B**) Dl is stronger expressed in TFs than in adjacent cap cells (brackets) in a *tj*^*z4735*^*/+* (control) ovary (A). Similarly, Dl is stronger expressed in the upper, Tj-negative portion of the extended TFs than in the adjacent Tj-positive cells (brackets) that represent transformed cap cells in the *tj*^*eo2*^*/tj*^*z4735*^ (*tj*^null^) ovary (B). (**C,D**) *E(spl)mß*-CD2 staining in the anterior niche is comparable between a *tj*^*z4735*^*/+* (control) ovary (C) and a *tj*^*z4735*^*/tj*^*eo2*^ (*tj*^null^) ovary (D). Genotypic markers: *bab-lacZ/+* (A,B), *E(spl)mß*-*CD2/+* (C,D). Anterior is up in all panels. Scale bars: 10 μm.
(TIF)

S1 Table Markers for cells of the GSC niche in the adult ovary. Relative expression level of cell markers in the adult GSC niche. n.d, not detected above background. *, References that describe the expression of markers in the GSC niche, including reference [82].
(DOCX)

S2 Table Markers for cells of the GSC niche in the prepupal ovary. Relative expression level of cell markers in the prepupal GSC niche. n.d, not detected above background. *, References for expression of a marker in the GSC niche.
(DOCX)

S3 Table Frequency of mosaic TFs. Clonal expression of *Act5C-Gal4* was used to drive expression of *UAS-tj*^*1(2)*^ in TF cells. *UAS-GFP* was used as a control.
(DOCX)
