# Supplementary figures and images for: Specification and spatial arrangement of cells in the germline stem cell niche of the Drosophila ovary depend on the Maf transcription factor Traffic jam
Source: PLoS Genet. 2017 May 19;13(5):e1006790. doi: 10.1371/journal.pgen.1006790 (PMC5459507; doi:10.1371/journal.pgen.1006790)

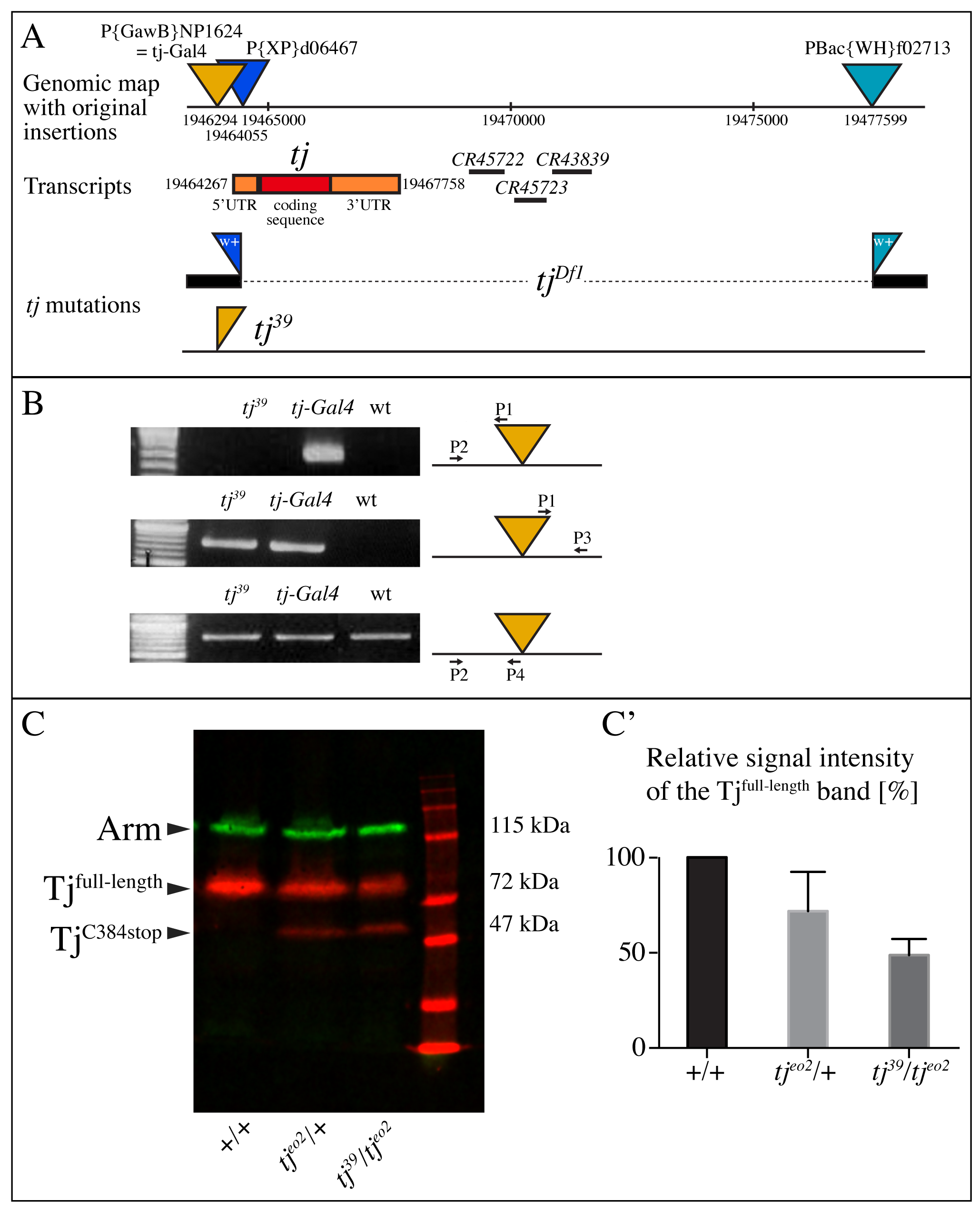

Supplement: S1 Fig — (A) Map of the genomic region encompassing the tj locus, showing the insertions used to generate the tjDf1 deletion in blue, and the insertion used to generate tj39 in yellow. tjDf1 deletes most of the transcription unit of tj, including the whole tj coding sequence and 3'UTR, and three predicted RNA coding genes. tj39 contains a partial P element in the promoter region of tj. (B) Analysis of the tj39 mutation by PCR shows that the 5' region of the tj-Gal4 insertion was excised but the flanking genomic regions remained unaffected. Target regions for primers P1-4 are indicated by arrows and listed in Material and Methods. (C) Immunoblot analysis of Tj proteins encoded by wild-type and tj mutant alleles in adult ovaries. The blot was probed with anti-Tj (red) and anti-Armadillo (Arm, green) antibodies. Wild-type Tj protein (Tjfull-length) runs at ~72 kDa, which is higher than expected based on its sequence (expected molecular weight: 54.3 kDa). tjeo2, which has a premature stop codon [36], produces a truncated non-functional protein (TjC384stop). The presence of the tj39 allele in tj39/tjeo2 ovaries caused a reduction in the amount of the Tjfull-length protein but not of the TjC384stop protein in comparison to tjeo2/+ ovaries. (C') Quantification of Tjfull-length protein, based on three immunoblots, including the one shown in (C), showing mean + s.d. The Tj signal was normalized to the Arm signal that was used as a loading control, and the Tj signal intensity from the wild-type (+/+) lane was set to 100%. (TIF) [file pgen.1006790.s001.tif]

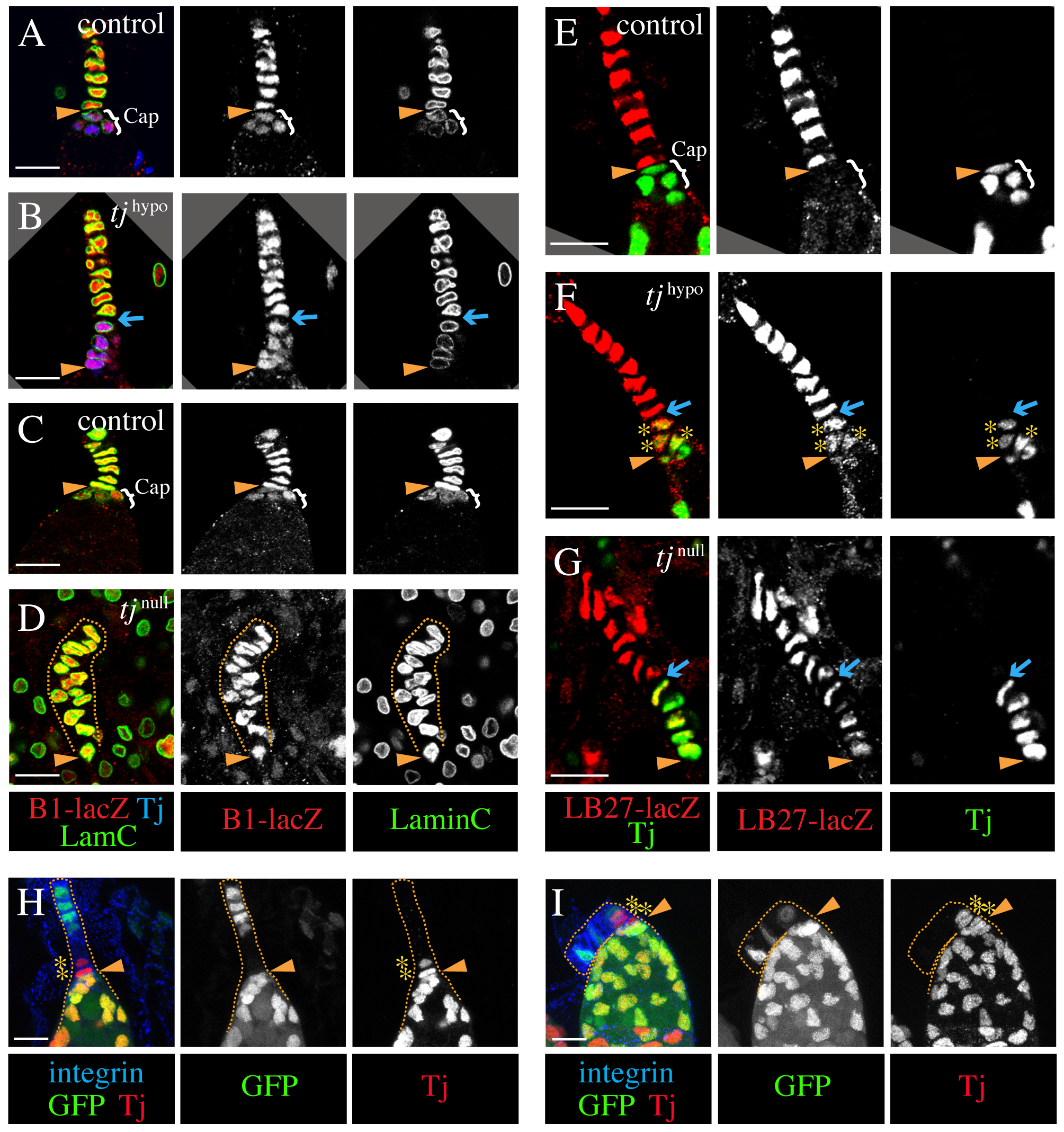

Supplement: S2 Fig — Images show the adult GSC niche. An arrowhead marks the TF/germarium boundary (A-I), a bracket the cap cell cluster (A,C,E), and an arrow the anterior-most Tj-positive cell in a TF (B,F,G). (A-D) B1-lacZ and LamC are strongly expressed in TF cells and weakly in Tj-positive cap cells in the controls (A,C) and in the tj39/tjeo2 ovary (tjhypo) (B). In contrast, both markers are strongly expressed throughout the extended TF (stippled line) of a tjeo2/tjeo2 mutant (tjnull) (D). (E-G) LB27-lacZ, which is exclusively detected in TF cells and absent from Tj-positive cap cells in the control (E), is sometimes seen in Tj-positive cap cells outside the germarium (asterisks) in a tj39/tjeo2 mutant (tjhypo) (F), and always found in the Tj-positive cells of the extended TF in a tjz4735/tjeo2 ovary (tjnull) (G). tjz4735 produces a detectable Tj mutant isoform. (H,I) tjnull mutant cell clones (homozygous for tjz4735) in the anterior niche. Images show projections of full Z-stacks of the GSC niches depicted in Fig 2H and 2I. Mosaic TFs contain tj mutant cells that lack GFP in the posterior portion. As these cells express Tj (mutant isoform), they represent transformed cap cells that are ectopically located in the TF (asterisks). Note that all escort cells in the vicinity of the cap cells express GFP. Genotypic markers: B1-lacZ/+ (A,B) or B1-lacZ (C,D), LB27-lacZ/+ (E-G), Ubi-GFP (H,I). Anterior is up in all panels. Scale bars: 10 μm. (TIF) [file pgen.1006790.s002.tif]

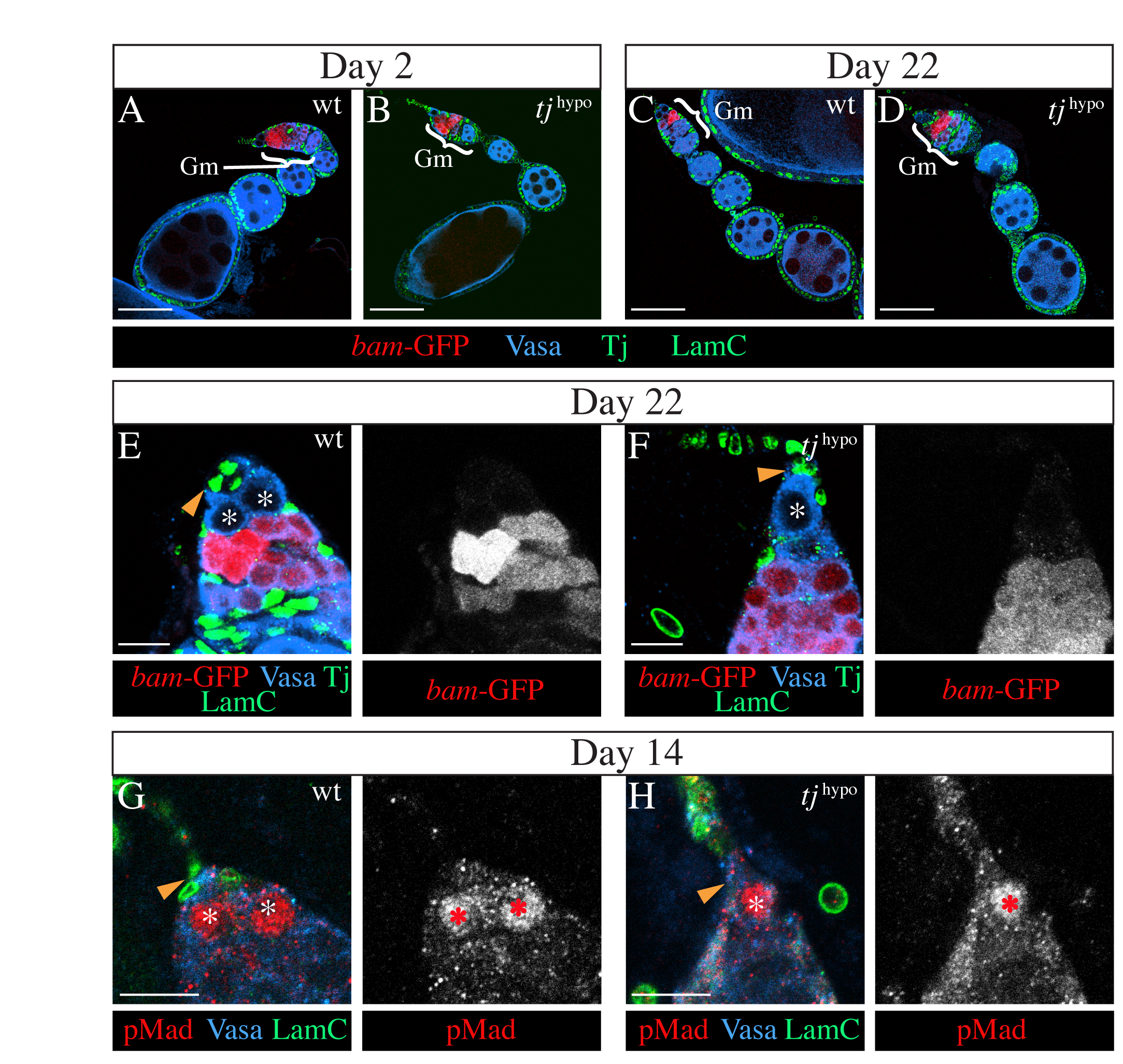

Supplement: S3 Fig — Vasa (blue) marks germline cells and LamC (green) labels TFs in all panels. Tj (green in A-F) marks cap, escort, and follicle cells, bam-GFP (red/white in A-F) marks early differentiating germ cells, and pMad (red/white in G,H) labels GSCs. The germarium-TF boundary is marked by an arrowhead (E-H). GSCs are marked by an asterisk. (A-D) Similar to the control (wt), tjeo2/tj39 ovarioles (tjhypo) contain a germarium (Gm) followed by a series of growing follicles in 2-day-old (A,B) and 3-week-old females (C,D), indicating the presence of at least one GSC. (E-H) The maintenance of two GSCs in a wild-type ovariole and one GSC in a tj mutant ovariole is indicated by the absence of bam-GFP (E,F) and the presence of pMad (G,H) in 3 and 2 week-old females, respectively. Genotypic marker: bam-GFP (A-F). Scale bars: 50 μm in A-D; 10 μm in E-H. (TIF) [file pgen.1006790.s003.tif]

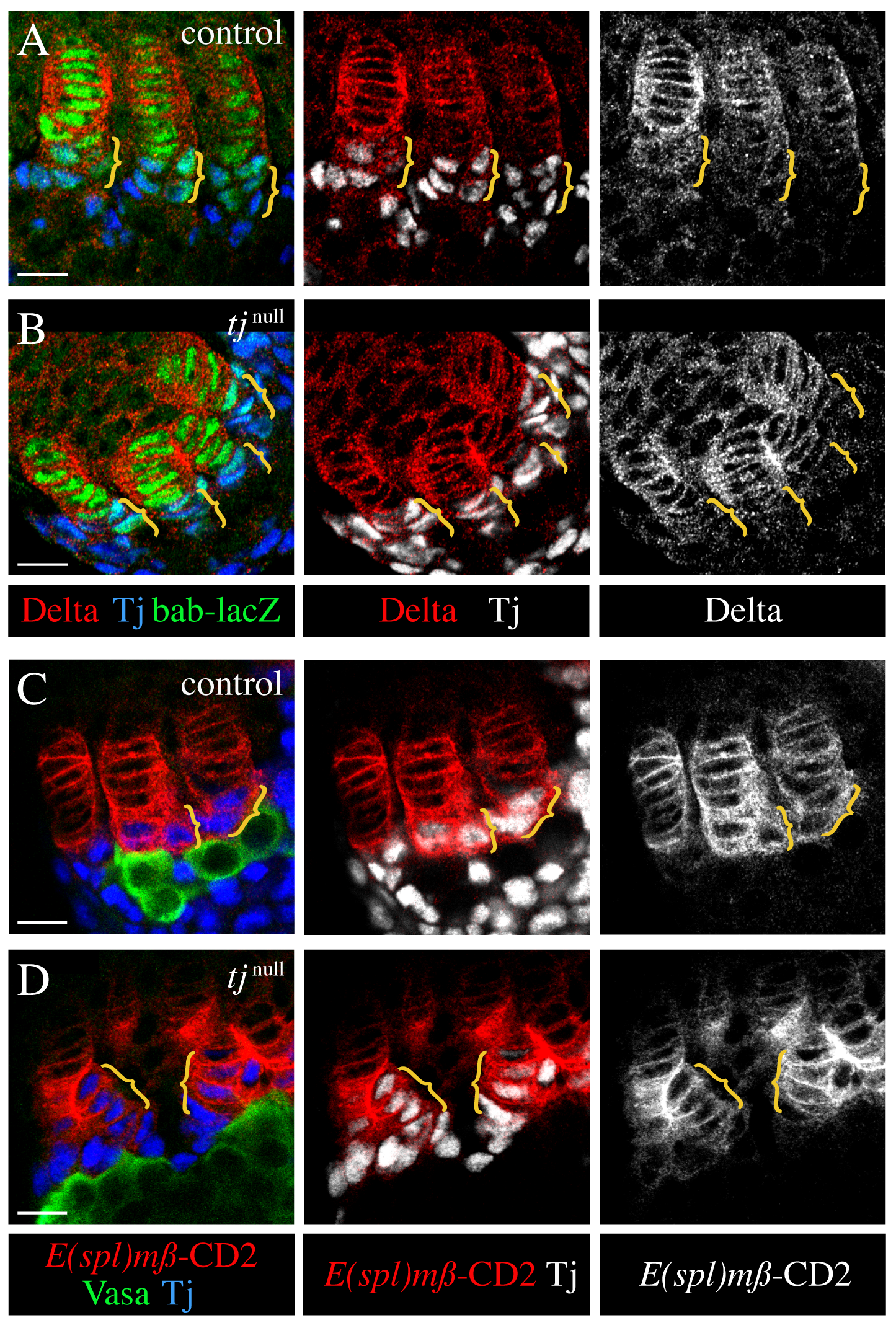

Supplement: S4 Fig — Images show the GSC niche in prepupal ovaries. Tj marks cap cells (brackets) and somatic cells that are intermingled with germ cells, bab-lacZ labels TF and cap cells, and Vasa labels germ cells. (A,B) Dl is stronger expressed in TFs than in adjacent cap cells (brackets) in a tjz4735/+ (control) ovary (A). Similarly, Dl is stronger expressed in the upper, Tj-negative portion of the extended TFs than in the adjacent Tj-positive cells (brackets) that represent transformed cap cells in the tjeo2/tjz4735 (tjnull) ovary (B). (C,D) E(spl)mß-CD2 staining in the anterior niche is comparable between a tjz4735/+ (control) ovary (C) and a tjz4735/tjeo2 (tjnull) ovary (D). Genotypic markers: bab-lacZ/+ (A,B), E(spl)mß-CD2/+ (C,D). Anterior is up in all panels. Scale bars: 10 μm. (TIF) [file pgen.1006790.s004.tif]
