# Supplementary material for: Specification and spatial arrangement of cells in the germline stem cell niche of the Drosophila ovary depend on the Maf transcription factor Traffic jam
Source: PLoS Genet. 2017 May 19;13(5):e1006790. doi: 10.1371/journal.pgen.1006790 (PMC5459507; doi:10.1371/journal.pgen.1006790)
Supplement: S1 Table — Relative expression level of cell markers in the adult GSC niche. n.d, not detected above background. *, References that describe the expression of markers in the GSC niche, including reference [82]. (DOCX) [file pgen.1006790.s005.docx]

**S1 Table. Markers for cells of the GSC niche in the adult ovary**

| **Marker** | **Terminal filament** | **Cap cells** | **Escort cells** | **Reference** * |
| --- | --- | --- | --- | --- |
| Tj | n.d. | strong | very strong | [36] |
| *1444-lacZ* | minimal | very strong | strong | [55] |
| Bab2 | weak | strong | weak |  |
| *bab-lacZ* | strong | strong | n.d. | [15] |
| *B1-lacZ* | strong | strong | n.d. | [56, 15] |
| Engrailed | strong | strong | n.d. | [82, 23] |
| LaminC | strong | weak | n.d. | [6, 25] |
| *LB27-lacZ* | strong | n.d. | n.d. | [16] |
