# Supplementary material for: Specification and spatial arrangement of cells in the germline stem cell niche of the Drosophila ovary depend on the Maf transcription factor Traffic jam
Source: PLoS Genet. 2017 May 19;13(5):e1006790. doi: 10.1371/journal.pgen.1006790 (PMC5459507; doi:10.1371/journal.pgen.1006790)
Supplement: S2 Table — Relative expression level of cell markers in the prepupal GSC niche. n.d, not detected above background. *, References for expression of a marker in the GSC niche. (DOCX) [file pgen.1006790.s006.docx]

**S2 Table. Markers for cells of the GSC niche in the prepupal ovary**

| **Marker** | **Terminal filament** | **Cap cells** | **Escort cells** | **Reference** * |
| --- | --- | --- | --- | --- |
| Tj | n.d. | strong | strong | [36, 22] |
| *1444-lacZ* | n.d. | strong | n.d. |  |
| *B1-lacZ* | strong | strong | n.d. |  |
| LaminC | only in the distal half of the TF | n.d. | n.d. | [22] |
| *LB27-lacZ* | strong | n.d. | n.d. | [16] |
| Engrailed | strong | strong | n.d. | [23] |
