# Supplementary material for: Specification and spatial arrangement of cells in the germline stem cell niche of the Drosophila ovary depend on the Maf transcription factor Traffic jam
Source: PLoS Genet. 2017 May 19;13(5):e1006790. doi: 10.1371/journal.pgen.1006790 (PMC5459507; doi:10.1371/journal.pgen.1006790)
Supplement: S3 Table — Clonal expression of Act5C-Gal4 was used to drive expression of UAS-tj1(2) in TF cells. UAS-GFP was used as a control. (DOCX) [file pgen.1006790.s007.docx]

**S3 Table. Frequency of mosaic TFs**

| **Transgene** | **Mosaic TFs/**  **total number of TFs** | **Mosaic TFs** [%] |
| --- | --- | --- |
| ***UAS-GFP*** | 69/170 | 40.6 |
| ***UAS-tj*** | 40/107 | 37.4 |
